# Virulence Determinants Are Required for Brain Abscess Formation Through *Staphylococcus aureus* Infection and Are Potential Targets of Antivirulence Factor Therapy

**DOI:** 10.3389/fmicb.2019.00682

**Published:** 2019-04-05

**Authors:** Ying Zheng, Weilong Shang, Huagang Peng, Yifan Rao, Xia Zhao, Zhen Hu, Yi Yang, Qiwen Hu, Li Tan, Kun Xiong, Shu Li, Junmin Zhu, Xiaomei Hu, Renjie Zhou, Ming Li, Xiancai Rao

**Affiliations:** ^1^Department of Microbiology, College of Basic Medical Sciences, Army Medical University, Key Laboratory of Microbial Engineering under the Educational Committee in Chongqing, Chongqing, China; ^2^Institute of Modern Biopharmaceuticals, School of Life Sciences, Southwest University, Chongqing, China; ^3^Department of Emergency, Xinqiao Hospital, Army Medical University, Chongqing, China

**Keywords:** *Staphylococcus aureus*, virulence determinant, brain abscess, ear colonization, bacteremia, antivirulence factor therapy

## Abstract

Bacterial brain abscesses (BAs) are difficult to treat with conventional antibiotics. Thus, the development of alternative therapeutic strategies for BAs is of high priority. Identifying the virulence determinants that contribute to BA formation induced by *Staphylococcus aureus* would improve the effectiveness of interventions for this disease. In this study, RT-qPCR was performed to compare the expression levels of 42 putative virulence determinants of *S. aureus* strains Newman and XQ during murine BA formation, ear colonization, and bacteremia. The alterations in the expression levels of 23 genes were further confirmed through specific TaqMan RT-qPCR. Eleven *S. aureus* genes that persistently upregulated expression levels during BA infection were identified, and their functions in BA formation were confirmed through isogenic mutant experiments. Bacterial loads and BA volumes in mice infected with *isdA, isdC, lgt, hla*, or *spa* deletion mutants and the *hla*/*spa* double mutant strain were lower than those in mice infected with the wild-type Newman strain. The therapeutic application of monoclonal antibodies against Hla and SpA decreased bacterial loads and BA volume in mice infected with Newman. This study provides insights into the virulence determinants that contribute to staphylococcal BA formation and a paradigm for antivirulence factor therapy against *S. aureus* infections.

## Introduction

Brain abscesses (BAs) are severe sequelae of central nervous system infections, and their prevention and treatment remains a major challenge in the medical field (Brouwer et al., 2014b). BAs are characterized as focally intracerebral lesions that are associated with the invasion of certain pathogens, the activation of resident parenchymal cells, and the influx and development of inflammatory cells into collections of pus surrounded by well-vascularized capsules (Kielian and Hickey, 2000). Intraparenchymal BA is a serious and potentially life-threatening condition and accounts for approximately 1 in 10,000 hospitalizations in the United States (Kielian and Hickey, 2000; Brouwer et al., 2014b). The treatment of BAs necessitates multiple approaches, including medical and surgical therapies. Patients with BAs should receive prompt empiric antibiotic therapy, and the surgical drainage of purulent material is necessary in most cases of BA (Brouwer et al., 2014b; Patel and Clifford, 2014). The most effective antimicrobial therapy for BAs, however, can be defined only after the infecting pathogen has been isolated (Brouwer et al., 2014b). The prevalence of antibiotic-resistant bacteria complicates the prevention and treatment of BAs. Thus, the development of alternative therapeutic strategies for BAs is urgently needed.

Although an etiological agent cannot be identified in some BA cases, the leading etiological agents of BA are streptococci and *Staphylococcus aureus* (Mathisen and Johnson, 1997). A recent meta-analysis revealed that 34% (2,000/5,894) and 18% (1,076/5,894) of culture-positive BA cases could be attributed to infections with *Streptococcus* and *Staphylococcus* spp., respectively (Brouwer et al., 2014a). *S. aureus* is a highly virulent and adaptable pathogen that is also a commensal species of human skin and nares (Tacconelli et al., 1998). Approximately 25% of individuals are permanently colonized with *S. aureus* and are therefore at a high risk of staphylococcal infections (Lowy, 1998; Cheng et al., 2009; Kobayashi et al., 2015). The emergence of methicillin-resistant *S. aureus* and vancomycin-intermediate *S. aureus* has aggravated the problem of bacterial drug resistance, which can result in the treatment failure of *S. aureus* infections with antibiotics (Hu et al., 2016). Antivirulence factor therapy has been proven as a powerful alternative intervention for *S. aureus* infections (McAdow et al., 2011; Chen et al., 2016). *S. aureus* possesses numerous virulence factors, including adhesins, toxins, and factors for escaping from host immune defenses (Zecconi and Scali, 2013; Zhang et al., 2017). The production of virulence factors by *S. aureus* may be strain-specific given the variability in the DNA sequences of *S. aureus* isolates (Baba et al., 2008). The wide range of outcomes related to *S. aureus* infections can be attributed to the ability of *S. aureus* to express different combinations of virulence factors in different infection sites (Zecconi and Scali, 2013). For example, coagulase and clumping factor A (ClfA) play specific roles in the pathogenesis of *S. aureus*-induced endocarditis (Moreillon et al., 1995). ClfA and ClfB act as key mediators in catheter-associated urinary tract infections caused by *S. aureus* (Walker et al., 2017). In a murine skin abscess model, the virulence of *S. aureus* strain Newman was impaired upon the deletion of the immune evasion molecule staphylococcal protein A gene (*spa*), the fibronectin-binding protein genes *fnbAB* and *clfA*, or the surface protein gene *sasF*; these genes, however, do not participate in the development of dermonecrosis (Kwiecinski et al., 2014). Renal abscess formation caused by *S. aureus* Newman involves the heme-scavenging factors IsdA and IsdB; the adhesins SdrD, Emp, and Eap; and the immune evasion factor SpA (Cheng et al., 2009).

In BA pathogenesis, *S. aureus* may originate continuously from a local source, such as the ears and nose, or from a hematogenously disseminated systemic infection (Patel and Clifford, 2014). Thus far, however, the virulence determinants involved in BA with *S. aureus* origins remain unclear. In this study, we used a murine model of ear colonization (EC) to represent the ecological niche of *S. aureus* and a murine model of bacteremia (BM) to mimic the hematogenous dissemination of *S. aureus* in BA. We compared the expression levels of 42 putative virulence determinants of *S. aureus* strains Newman and XQ in the mouse models of BA, EC, and BM through reverse transcription-quantitative real-time polymerase chain reaction (RT-qPCR) analysis. We confirmed alterations in gene expression levels through specific TaqMan RT-qPCR. We validated the functions of persistently upregulated *S. aureus* virulence genes in BA formation through isogenic mutant experiments. We found that the therapeutic application of monoclonal antibodies (mAbs) against Hla and SpA inhibited BA formation in mice infected with the wild-type *S. aureus* Newman strain. Our data provide novel insights into the virulence determinants that contribute to staphylococcal BA formation and will help guide the development of an antivirulence paradigm for the treatment of *S. aureus*-induced BA.

## Materials and Methods

### Bacterial Strains and Cultures

All bacterial strains and plasmids used in this study were listed in Supplementary Table S1. *S. aureus* Newman (NCTC 8178, ST1/*agr* I) was isolated from a throat swab from a patient suffered from secondarily infected tubercular osteomyelitis (Duthie and Lorenz, 1952; Missiakas and Schneewind, 2013). *S. aureus* XQ (ST121/*agr* IV) is a highly virulent strain isolated from a 16-year-old juvenile, whose onset was a skin wound then deteriorated to a lethal *S. aureus* sepsis with mental disorder (Rao et al., 2015; Liu et al., 2018). *S. aureus* strains were cultured on tryptic soy agar (Oxford, the United Kingdom) or in tryptic soy broth (TSB, Oxford) at 37°C with shaking at 200 rpm overnight, then diluted 1:100 in TSB for culture or harvested in mid-log phase by centrifugation for inoculation. After washed twice by sterile PBS, the pellets were resuspended in PBS for use. *Escherichia coli* strain DH5α was cultivated in Luria Broth (LB) medium (Oxoid) supplemented with proper antibiotics for maintaining certain plasmids.

### Construction of Murine Models Through *S. aureus* Infection

BALB/c-nu/nu mice (female, 6 weeks old) and C57BL/6 mice (6–8 weeks of age) were purchased from the Experimental Animal Centre of the Army Medical University (AMU). This study was carried out in accordance with the recommendations of the Regulations for the Administration of Affairs Concerning Experimental Animals approved by the State Council of People’s Republic of China. The protocol was approved by the local ethics board at the Laboratory Animal Welfare and Ethics Committee of Army Medical University (#SYXK-PLA-20120031).

For BA model, female C57BL/6 mice (*n* = 10) were anesthetized with 1% pentobarbital sodium (50 mg/kg, Sigma–Aldrich, the United States). A middle longitudinal incision was made along the scalp from ear to eye, exposing the frontal sutures. The mouse head was fixed in a stereotactic frame and drilled a hole near the bregma (1 mm rostral, 2 mm lateral to the right of bregma). A suspension of 5 μl prepared *S. aureus*-encapsulated agarose beads (1 × 10^5^ CFU) was injected 3 mm deep into the brain by microsyringe through the hole, waiting for 2 min after the infusion to minimize efflux. The pure agarose beads were injected and served as blank control. Pull out the needle slowly, and suture the skin as described (Baldwin and Kielian, 2004; Bloch et al., 2005). Three days after infection, mice were euthanized. The brain tissues were immersed in RNAlater (Qiagen, Germany), incubated at 4°C overnight for RNA extraction.

For EC model, both ears of BALB/c-nu/nu mice (*n* = 10) were colonized with 1 × 10^8^ CFU of *S. aureus* strain Newman or XQ. Mice were kept in individually ventilated cages to prevent contamination. Seventy-two hours later, the external acoustic meatus of each mouse were wiped by wet swabs with 100 μl RNAlater (Qiagen) for sample collection, and then the samples were stored at 4°C overnight before RNA extraction.

For BM model, C57BL/6 mice (*n* = 10) were injected with 1 × 10^8^ CFU of *S. aureus* strain Newman or XQ in 100 μl PBS through tail vein injection. One hour after injection, mouse blood was collected by heart punctuation after anesthetized with 1% pentobarbital sodium, and immediately mixed with 10 volumes of cold EL buffer (Qiagen). The mixture was then incubated on ice for 10 min with intermittent vortex mixing to split the erythrocytes thoroughly. The mixture of leukocytes and bacteria was harvested by centrifuging at 4,500 × *g* for 10 min at 4°C. The pellets were resuspended in an equal blood volume of ice-cold RNase-free water, pooled into two volumes of RNAprotect Bacteria Reagent (Qiagen). Finally, *S. aureus* cells were gathered by centrifugation and resuspended in TriPure (Roche Life Science, Switzerland).

Three independent experiments with 10 mice each were performed as biological repetitions.

### Total RNA Extraction

The ear swabs and centrifuged pellets were resuspended in 1.2 ml of pre-chilled buffer RLT from RNeasy Mini Kit (Qiagen) supplemented with 1% β-mercaptoethanol (Sigma–Aldrich). The mixture was transferred to lysing matrix B tubes (MP Biomedicals, the United States), then followed by mechanical disruption in a Mini Bead Beater (Biospec, the United States) for three cycles with 40 s each and 2 min interval incubation on ice. After centrifugation at 13,500 × *g* for 5 min, the supernatant was transferred to the RNeasy Mini Kit column for the following procedures of RNA extraction according to the manufacturer’s instructions. DNase (Qiagen) treatment was performed with optional on-column DNA digestion. Finally, RNA was eluted with RNase-free water as described (Chaves-Moreno et al., 2016).

The RNA extraction procedures of BM and BA samples followed the standard phenol/chloroform/isopropanol protocol with some modifications. The specimen was disrupted by bead-milling submerged in TriPure as the aforementioned mechanical disruption process (Jenkins et al., 2015). The RNA pellet was resuspended in RNase-free water, digested with RNase-free DNase (Promega, the United States) for 1 h. Afterward, the TriPure extraction and DNase digestion procedures were repeated once to eliminate residual DNA. Finally, clean up of RNA was performed by using the RNeasy Mini Kit, and the eluting RNA was diluted in RNase-free water for reverse transcription.

### RT-qPCR

Reverse transcription was performed by SuperScript III cDNA Synthesis Kit (Invitrogen Corporation, the United States) following the manufacturer’s recommendations. The 16S rRNA gene was used as the reference gene according to the observation of its expression variation never exceeded twofold in different growth conditions (Supplementary Figure S1). SYBR^®^Green qPCR was performed using SsoAdvanced Universal SYBR^®^Green Supeimix (Bio-Rad, the United States) with the cDNA as templates in the 7500 Real-Time PCR System (Applied Biosystems, the United States). For TaqMan qPCR, probes for the candidate genes were designed and were 5′-FAM (reporter) and 3′-TAMRA (quencher) labeled (Supplementary Table S2). TaqMan qPCR was carried out using Platinum Taq DNA Polymerase mix (Thermo Fisher Scientific, the United States) with standard cycling protocols, and data were analyzed using StepOne Software (Applied Biosystems, the United States). All primer pairs (listed in Supplementary Table S2) were identical for SYBR^®^Green and TaqMan-based qPCR to ensure consistency as much as possible for the same gene between the two methods.

### Construction of In-frame Gene Deletions

To construct isogenic markerless knockout mutants of *S. aureus* Newman, the in-frame gene deletions were performed by allelic replacement using the temperature-sensitive *E. coli* and *S. aureus* shuttle vector pBT2 as previously described (Supplementary Figure S2A; Yuan et al., 2018). Each mutant of interest was verified by PCR amplification with certain primers listed in Supplementary Table S2 and DNA sequencing.

### Bacterial Load Counting in Mouse Brain

Five days after intracranial inoculation with *S. aureus* Newman or mutant strains, mice were implemented euthanasia. The whole brain was taken and homogenized with 1 ml of 1% Triton X-100 (Sigma-Aldrich) in PBS, then serially diluted (10-fold) for the enumeration of viable bacteria by plating on tryptic soy agar and cultured for 24 h at 37°C.

### Histological Examination and Brain Abscess Volume Computation

Five days after infection, mice were euthanized and the brain was removed and fixed in 4% paraformaldehyde. The infiltrated brain tissues were then embedded in paraffin. Serial sections in 4 μm thickness were sampled every 100 μm throughout the brain and stained with hematoxylin and eosin (H&E). Each section was examined and photographed under bright field microscopy (BX52, Olympus, Japan) at 2× or 40× objective magnification. The BA volumes were computed by Image J (NIH, the United States) as described (Bloch et al., 2005).

### mAb Therapy for Murine Brain Abscess

Anti-Hla and anti-SpA mAbs of murine origin (BALB/c) were kindly provided by Prof. Hao Zeng (AMU). In the treatment group, a combination of modified anti-Hla mAbs (including two clones of 4D11 and 7G4) and anti-SpA mAbs (including 2H4 and 8C3 clones) diluted in 100 μl PBS was injected intraperitoneally (i.p.) into C57BL/6 mice (5 mg/kg). For the control group, the same volume of PBS was given instead. BA models were established 24 h after i.p. administration. Five days later, the two groups of BA mice were euthanized for bacterial load counting and BA volume calculation as aforementioned.

### Three-Dimensional Reconstruction of Brain Abscess

Histological sections of mouse brain infected with *S. aureus* Newman pretreated with or without mAbs against Hla and SpA were prepared by sampling every 100 μm and photographed in sequence by microscopy at 20× magnification. The visible brain structures including the abscess in each section were circled using the histological sections slide by slide with Amira software (version 5.2.2) and the three-dimensional (3-D) structure of the infected murine brain was reconstructed by Cinema 4D R18 (Ruthensteiner and Hess, 2008).

### Statistical Analysis

Statistical analysis of results was carried out using GraphPad Prism 7. Unpaired two-tailed Student’s *t*-test was used to treat samples between two groups, and Mann–Whitney test was used for testing multiple groups. Each experiment was carried out at least thrice. Results are presented as mean ± standard deviations (SD), and a *P*-value <0.05 was considered statistically significant. ^∗^*P* < 0.05, ^∗∗^*P* < 0.01, ^∗∗∗^*P* < 0.001, and ns represented no significance.

## Results

### Establishment of Mouse BA Models and RT-qPCR Analysis of the Expression Levels of *S. aureus* Virulence Genes

**FIGURE 1:**
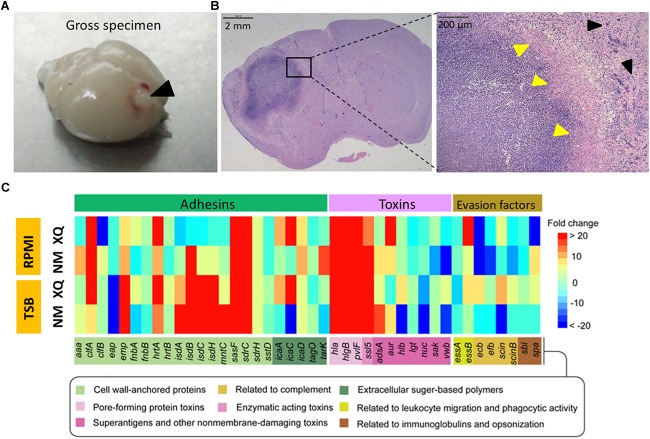
Comparison of the expression levels of virulence genes under *in vivo* and *in vitro* conditions. **(A)** Mouse BA model induced by *S. aureus*. Gross view of an abscess prominent in the brain collected from a model mouse at 5 days after infection with the Newman strain (black arrowhead). **(B)** Hematoxylin and eosin (H&E) staining and examination of brain section collected from a model mouse at 5 days post infection revealed a well-circumscribed abscess at low magnification (20×, left panel) and a defined abscess with neutrophil and macrophage infiltration and abscess capsule margin (yellow arrowheads) in the normal brain tissue (black arrowheads) at high magnification (400×, right panel). **(C)** A heatmap showed fold changes in the expression levels of virulence genes in BA, TSB, and RPMI. Results are presented as mean fold of upregulation (shades of red) and downregulation (shades of blue) in three separate samples after detection by SYBR^®^Green RT-qPCR (see scale bar). Fourty-two virulence genes were divided into three groups (adhesins, toxins, and immune evasion factors) and their functions were indicated after Gene Ontology analysis.

We used the *S. aureus* Newman and XQ strains to establish mouse models of BA. *S. aureus* Newman (ST1/*agr* I) has been widely used to construct animal models of staphylococcal diseases because of its stable *agr* phenotype (Moreillon et al., 1995; Cheng et al., 2009; Missiakas and Schneewind, 2013; Zecconi and Scali, 2013; Kwiecinski et al., 2014; Zhang et al., 2017). *S. aureus* XQ (ST121/*agr* IV) is a highly virulent clinical strain collected by our laboratory (Rao et al., 2015). The complete genome sequences of the Newman and XQ strains have been uploaded to GenBank with accession numbers of AP009351.1 and CP013137.1, respectively (Baba et al., 2008; Liu et al., 2018). These strains express strain-specific virulence factors but also share numerous common virulence determinants (Supplementary Figure S3). The clinical course developed by C57BL/6 mice after intracranial injection with *S. aureus*-encapsulated agarose beads was similar to that developed by previously reported BA models (Baldwin and Kielian, 2004; Bloch et al., 2005). Gross specimens revealed the formation of focal BAs in the parenchymal area of the murine brain (Figure 1A). Histochemical staining (H&E) and examination of infected brains at days 5 post infection revealed well-circumscribed abscesses with neutrophil and macrophage infiltration and drastic mass effects and midline shifts (Figure 1B). The pure agarose beads without *S. aureus* (blank control) injected mice were unable to form BA, only a needle passway remained (Supplementary Figure S4).

Bacterial gene expression profiles under *in vitro* conditions differ from those under *in vivo* conditions (Jenkins et al., 2015). We applied SYBR^®^Green RT-qPCR to determine variations in *S. aureus* gene expression in response to environmental changes. We used TSB and RPMI medium 1640 (RPMI), the typical media used for culturing bacteria and eukaryotic cells, respectively, to represent *in vitro* conditions and establish baseline conditions (Jenkins et al., 2015). We utilized the mouse BA model to represent the *in vivo* environment. The 16S rRNA gene served as an internal control for the normalization of gene expression level because its expression in Newman and XQ strains under various *in vitro* growth conditions varied by less than twofold (Supplementary Figure S1). We performed a control PCR assay based on the *Pseudomonas aeruginosa pcrV* gene in a plasmid to exclude possible copurified contaminants. We amplified the *pcrV* gene with similar efficiency from all samples. This result suggests that none of the purified cDNA samples affected PCR efficiency (Supplementary Figure S5). The expression levels of 42 putative virulence determinants of Newman and XQ strains (Supplementary Table S3) were determined and normalized to those of the 16S rRNA gene. We categorized the 42 virulence determinants as adhesins, toxins, or immune evasion factors (Zecconi and Scali, 2013; Becker, 2018). The fold changes in the expression levels of each virulence gene in BA versus those in TSB or RPMI (baseline) were calculated and shown as a heatmap (Figure 1C). In at least one strain, the expression levels of most adhesin genes were upregulated from 2.2-fold to 32,000-fold during the transition from external growth to internal BA formation. In both strains, the transcription levels of the adhesion genes *clfA, emp, hrtA, sasF*, and *sdrC* increased under *in vivo* conditions relative to those under *in vitro* culture conditions. In both strains, the expression levels of *isdA, isdB, isdC, isdH*, and *mntC*, which are all adhesins related to metal cation transport, were upregulated under *in vivo* conditions relative to those under *in vitro* culture conditions. These results, however, did not hold true for XQ in RPMI. During BA formation, the expression levels of the typical pore-forming toxin genes *hla, hlgB, lukF-PV* (*pvlF*), and *ssl5* consistently increased, whereas those of genes encoding immune evasion factors varied (Figure 1C).

### Differential Expression Patterns of Virulence Determinants in Murine Models of BA, EC, and BM

**FIGURE 2:**
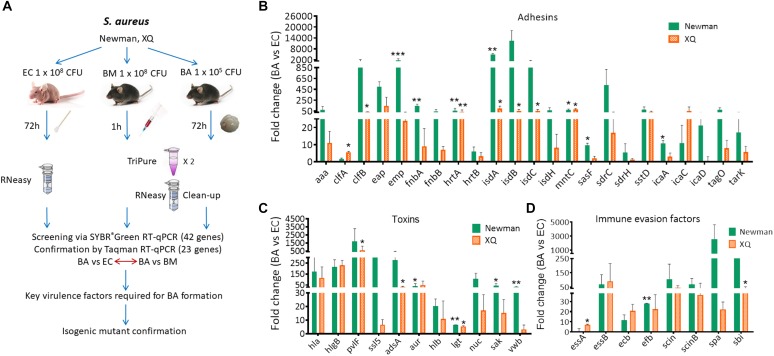
RT-qPCR detection of the expression levels of virulence determinants in the murine models of BA and EC. **(A)** Schematic of the experiment performed to identify key virulence determinants that contribute to the formation of BA induced by *S. aureus*. BALB/c-nu/nu or C57BL/6 mice were infected with the indicated dose of *S. aureus* Newman or XQ. Samples were then collected at 1 or 72 h after bacterial infection and subjected to SYBR^®^Green RT-qPCR for the detection of 42 virulence genes. The 23 genes of interest were further confirmed through TaqMan RT-qPCR analysis. The expression patterns of *S. aureus* genes in the BA model were compared with those in the EC and BM models. The functions of screened genes were further identified through isogenic mutant experiments. SYBR^®^Green RT-qPCR detection of virulence profiles. **(B)** Adhesin genes, **(C)** Toxin genes, and **(D)** Immune evasion factor genes in the mouse models of BA and EC. The transcript levels of genes were normalized to those of the 16S rRNA gene, and results for the BA model were compared with those for the EC model. The *S. aureus* strains Newman (green bars) and XQ (orange bars) were analyzed. Results are from three independent experiments with 10 animals/experiment. ^∗^*P* < 0.05, ^∗∗^*P* < 0.01, ^∗∗∗^*P* < 0.001 (Student’s *t*-test).

**FIGURE 3:**
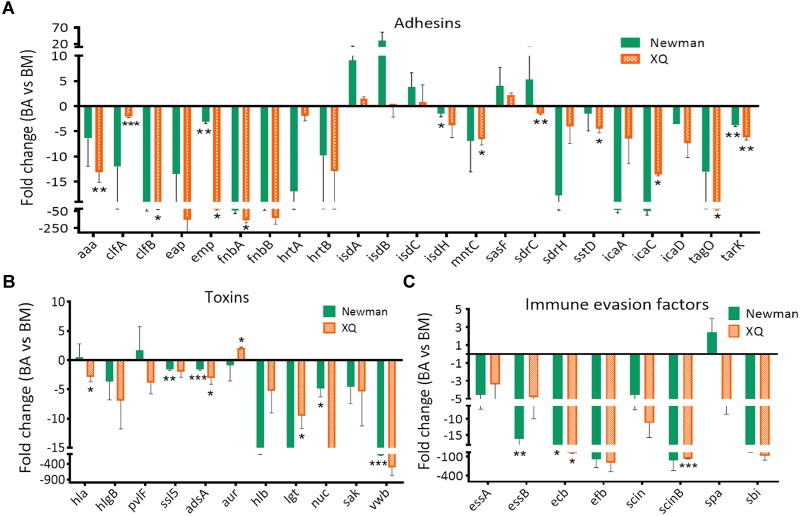
Variations in the expression patterns of virulence determinants in murine models of BA and BM. The transcript levels of genes were normalized to those of the 16S rRNA gene, and results for the BA model were compared with those for the BM model. The *S. aureus* strains Newman (green bars) and XQ (orange bars) were analyzed. Results are from three independent experiments with 10 animals/experiment. ^∗^*P* < 0.05, ^∗∗^*P* < 0.01, ^∗∗∗^*P* < 0.001. **(A)** Adhesin genes, **(B)** toxin genes, and **(C)** immune evasion factor genes.

Skin-colonizing and hematogenously disseminated *S. aureus* are major sources of brain infections after traumatic brain injuries and neurosurgical procedures (Baldwin and Kielian, 2004; Bloch et al., 2005; Patel and Clifford, 2014). We used BALB/c-nu/nu mice instead of C57BL/6 mice to establish the EC model because bacteria were eliminated from the external auditory skin of C57BL/6 mice within 1 week after inoculation with *S. aureus* (Katayama et al., 2013). We successfully developed the mouse model of *S. aureus*-induced BM by intravenously injecting *S. aureus* Newman or XQ into C57BL/6 mice. We used the EC model to represent skin colonization with commensal *S. aureus* under normal physical conditions and the BM model to represent systemic infection. We hypothesized that the virulence profiles of *S. aureus* in the BA model differ from those in the EC and BM models. The schematic of the experiment that we performed to identify the potential virulence determinants involved in BA formation is shown in Figure 2A. SYBR^®^Green RT-qPCR was applied to investigate the changes in the expression levels of the 42 putative virulence genes (Supplementary Table S3) in the EC and BM models. As indicated in Figure 2B–D, the expression levels of all 42 selected genes of the Newman and XQ strains, except for those of *clfA* and *essA* of the Newman strain and *icaD* of the XQ strain, increased by more than twofold in the BA model relative to those in the EC model. Among these genes, the expression levels of 11 adhesion genes (*clfA, clfB, emp, fnbA, hrtA, isdA, isdB, isdC, mntC, sasF*, and *icaA*), 6 toxin genes (*pvlF, adsA, aur, lgt, sak*, and *vwb*), and 3 immune evasion factor genes (*essA, efb*, and *sbi*) of at least one strain were significantly upregulated (*P* < 0.05). Similarly, the expression levels of all selected genes of the Newman and XQ strains, except for those of *sasF* of the XQ strain, increased by more than twofold in the BM model relative to those in the EC model (Supplementary Figure S6). These results indicate that the brain and blood represent more challenging infectious status than skin colonization for *S. aureus*. The expression levels of 78.6% (33/42) of the detected genes of the Newman and XQ strains decreased by more than twofold in the BA model relative to those in the BM model (Figure 3). The expression levels of the adhesin genes *emp* and *tarK*, toxin gene *adsA*, and immune evasion gene *ecb* of both strains were significantly downregulated in the BA model relative to those in the BM model (*P* < 0.05). The expression levels of *isdA, isdB, isdC, sasF, sdrC, hla, pvlF, aur*, and *spa* of at least one strain were upregulated in the BA model relative to those in the BM model. These genes have crucial roles in the development of BAs through *S. aureus* infection (Figure 3).

### TaqMan RT-qPCR and Isogenic Mutant Analyses of Virulence Determinants That Contribute to BA Formation by *S. aureus*

**FIGURE 4:**
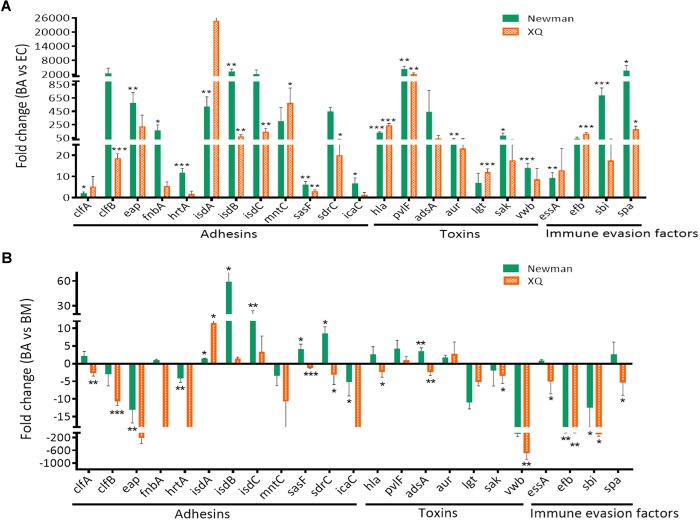
Confirmation of virulence gene expression through TaqMan RT-qPCR. The TaqMan probes designed for the virulence genes were listed in Supplementary Table S2. The transcript levels of genes were normalized to those of the 16S rRNA gene, and results for the BA model were compared with those for the EC **(A)** and BM **(B)** models. The *S. aureus* strains Newman (green bars) and XQ (orange bars) were analyzed. Results are from three independent experiments with 10 animals/experiment. ^∗^*P* < 0.05, ^∗∗^*P* < 0.01, ^∗∗∗^*P* < 0.001.

The TaqMan approach exhibits high reproducibility, specificity, and sensitivity, as well as low sensitivity to genomic DNA contaminants in RNA samples (Alvarez and Doné, 2014). We applied the TaqMan RT-qPCR approach to confirm the expression patterns of the genes that were identified through SYBR^®^Green RT-qPCR. We selected nine genes (*isdA, isdB, isdC, sasF, sdrC, hla, pvlF, aur*, and *spa*) that were upregulated in the BA model relative to those in the BM model (Figure 3). We also selected 14 genes (*clfA, clfB, emp, fnbA, hrtA, mntC, icaA, adsA, lgt, saK, vwb, essA, efb*, and *sbi*) with expression levels that were significantly upregulated in at least one strain in the BA model relative to those in the EC model (Figure 2, *P* < 0.05) but were downregulated in the BA model relative to those in the BM model. We found that the expression levels of all tested virulence genes, except for those of *hrtA* and *icaC* of the XQ strain, increased by at least twofold (Figure 4A and Supplementary Figure S7). Among the detected genes, the expression levels of *isdB, sasF, hla, pvlF*, and *spa* of both strains were significantly upregulated in the BA model relative to those in the EC model (*P* < 0.05). The expression levels of other 18 genes, except for *adsA*, of at least one strain were markedly upregulated in the BA model relative to those in the EC model (Figure 4A). Notably, the expression levels of 13 selected genes (56.5%, 13/23) were upregulated. Six genes of at least one strain were significantly enriched (*isdA, isdB, isdC, sasF, sdrC*, and *adsA*) in the BA model relative to that in the BM model (Figure 4B, *P* < 0.05). These results are similar to those detected through SYBR^®^Green RT-qPCR (Figure 3).

**FIGURE 5:**
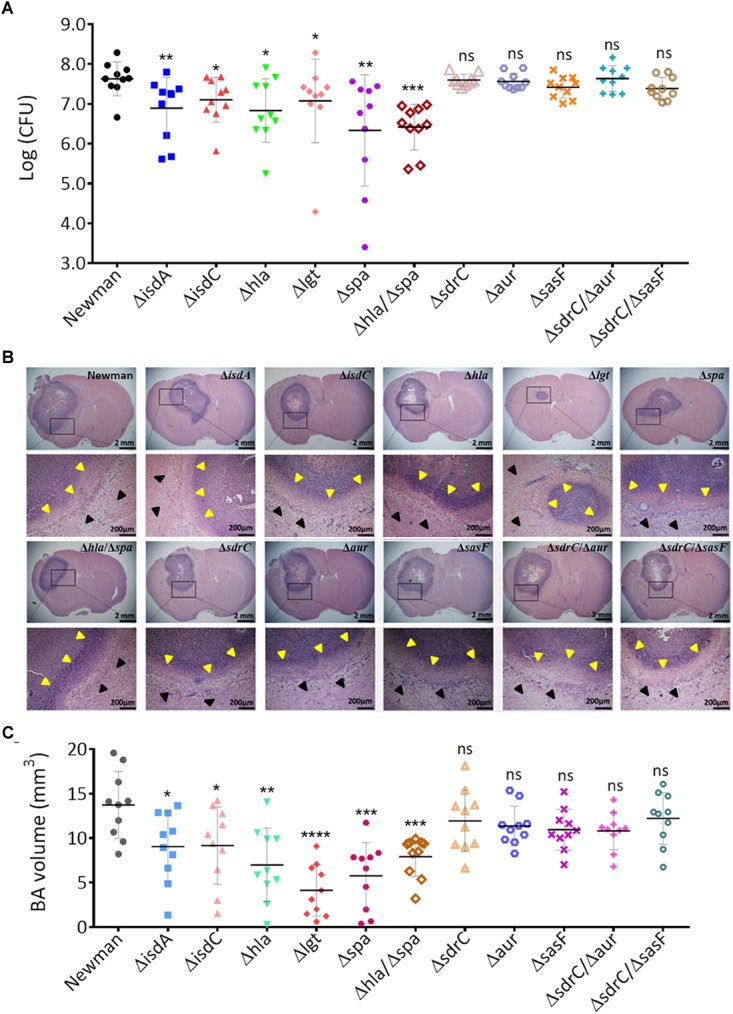
Isogenic mutant analysis of virulence determinants that contribute to BA formation through *S. aureus* infection. **(A)** CFU enumeration of wild-type Newman and mutant strains in the BA model. Groups of 10 mice each were challenged intracranially with 5 μl of agarose beads encapsulated with 1 × 10^5^ CFU of *S. aureus*. At 5 days after infection, the number of the viable organisms associated with BA was determined by quantitative culture. Titers are expressed as the mean log (CFU) per mouse in the brain homogenate. **(B)** H&E-stained brain sections collected from mice at 5 days post infection with wild-type Newman or mutant strains. For each strain, sections collected from a representative mouse brain revealed a focal intracerebral lesion (20×, top panel) and a well-defined abscess surrounded by a clear boundary (yellow arrows) within the normal brain tissue (black arrows, 400×, bottom panel). **(C)** BA volume calculation of mice infected with the wild-type Newman or mutant strains. The abscess areas of each brain section slide were evaluated.

To confirm that the above virulence determinants are required in BA formation by *S. aureus*, we attempted to construct isogenic mutants by using five genes (*isdB, sasF, hla, pvlF*, and *spa*) of both strains with expression levels that were significantly upregulated in the BA model relative to those in the EC model (Figure 4A, *P* < 0.05), three consistently upregulated genes (*isdA, isdC*, and *aur*) of both strains with expression levels that were upregulated in the BA model relative to those in the BM model, and two genes (*sdrC* and *adsA*) of the Newman strain with expression levels that were upregulated in the BA relative to those in the BM model (Figure 4B). We also selected the *lgt* gene. The expression level of the *lgt* gene of XQ was significantly upregulated in the BA model relative to that in the EC model (*P* < 0.001), whereas that of the *lgt* gene of both strains was slightly downregulated in the BA model relative to that in the BM model (Figure 4). Given that *S. aureus* XQ is a clinical isolate and is difficult to genetically manipulate (Liu et al., 2018), we constructed isogenic mutants with a Newman background. Eight single-gene mutants were successfully constructed through homologous DNA recombination without any selection markers (Supplementary Figure S2A) and verified through PCR amplification and DNA sequencing (Supplementary Figures S2B–I). The unsuccessful deletion of *isdB, pvlF*, and *adsA* may attribute to problems with the applied technique (Yuan et al., 2018). We also constructed three double-gene mutants (Δ*hla*/Δ*spa*, Δ*sdrC*/Δ*aur*, and Δ*sdrC*/Δ*sasF*) to evaluate the synergism between the selected genes. BA models were separately established with the wild-type Newman and mutant strains. The brain bacterial loads of mice infected with the mutant strains were quantified and compared with those of mice infected with the wild-type Newman strain. The colony-forming units (CFUs) of the *isdA, isdC, lgt, hla*, or *spa* deletion mutants and those of the *hla*/*spa* double mutant were lower than those of the wild-type Newman strain, whereas those of the *sdrC, aur, sasF, sdrC*/*aur*, or *sdrC*/*sasF* knockout mutants did not differ from those of the wild-type Newman strain (Figure 5A).

Hematoxylin and eosin-stained brain sections collected from mice at 5 days after infection with the wild-type Newman or mutant strains exhibited typical BA formations that were characterized by focal intracerebral lesions with clear boundaries (Figure 5B). We calculated the BA volume through ImageJ software as previously described (Bloch et al., 2005). In accordance with the bacterial load results, the volumes of BAs resulting from infection by the *isdA, isdC, lgt, hla*, or *spa* deletion mutants and those from infection by the *hla*/*spa* double mutant significantly decreased relative to those of BAs resulting from infection by the wild-type Newman strain (*P* < 0.05), whereas of BAs resulting from infection with *sasF, sdrC, aur, sdrC*/*sasF*, or *sdrC/aur* mutants and wild-type strain negligibly differed (Figure 5C). Taken together, these data indicate that the *isdA, isdC, lgt, hla*, and *spa* genes are required for BA formation through *S. aureus* infection. Potential antivirulence strategies that target these specific determinants may be developed for the treatment of *S. aureus*-induced BA infections.

### Mitigation of Staphylococcal BA Formation in Mice by Antivirulence Treatment

**FIGURE 6:**
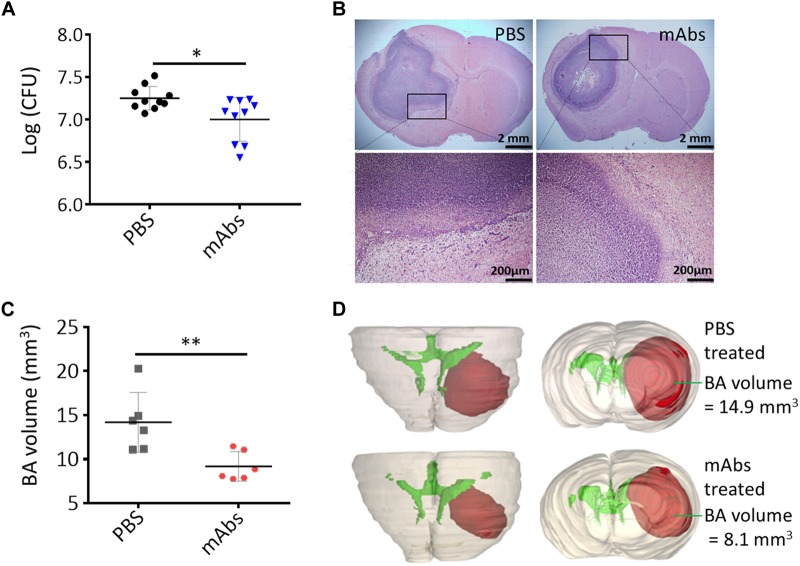
Synergistic anti-BA activities of mAbs against *S. aureus* Hla and SpA. **(A)** Bacterial loads. A group of 10 mice were each pretreated i.p. with mAbs against Hla and SpA (10 μg/kg) and then challenged intracranially with agarose beads encapsulated with 1 × 10^5^ CFU of *S. aureus* wild-type Newman strain. The PBS-treated mice served as negative control. Five days after infection, the number of viable organisms associated with BA was determined by quantitative culture. Titers are expressed as the mean log (CFU) per mouse of brain homogenate, ^∗^*P* < 0.05. **(B)** Histological examination showed BA lesions with similar characteristics developed under treatment with PBS and mAbs. **(C)** BA volumes. BA volume of each mouse was calculated based on the 3-D structure of BA constructed by using BA histological section slides. *n* = 6, **^∗∗^***P* < 0.01. **(D)** Three-dimensional reconstruction of the representative mouse brain showed the location and volume of BA after PBS or mAbs treatment. See 3-D animation in Supplementary Figures S8, S9.

We tested the anti-BA activities of mAbs against certain virulence determinants to determine the potential of their potential as antivirulence targets in the treatment of BA caused by *S. aureus* infection. Different mAbs that target different epitopes may exert synergistic protective effects when coadministered (Chow and Casadevall, 2012). We administered mAbs against Hla and SpA to C57BL/6 mice through intraperitoneal injections. On the day after mAb treatment, we cerebrally infected mice with the wild-type Newman strain to establish BA infections. Bacterial load and BA volume were quantified at 5 days postinfection. As shown in Figure 6A, the viable bacterial counts in the murine BA model treated with mAbs against Hla and SpA significantly decreased relative to those in the model treated with PBS (*P* < 0.05). Although histological examinations revealed that BA lesions with similar characteristics developed under treatment with PBS and mAbs (Figure 6B), BA volumes significantly decreased under treatment with mAbs (Figure 6C, *P* < 0.01). This result was also confirmed through 3-D modeling (Figure 6D). Overall, these results show that treatment with specific antibodies against virulence determinants may reduce the pathogenicity of *S. aureus*-induced BA and thus represents an alternative strategy for the clinical treatment of brain infections caused by *S. aureus*.

## Discussion

*Staphylococcus aureus* can colonize and infect various sites of the human body because of its ability to employ different sets of virulence factors during adaptation to specific niches (Zecconi and Scali, 2013). Adhesin genes, such as *clfB, sdrC, sdrD, sasG*, and *tarK*, have important roles in the commensal lifestyle of *S. aureus* (Burian et al., 2010a,b). The genes *clfA, tagO*, and *fnbA* have been implicated in the development of *in vivo* organ infection (Weidenmaier et al., 2005; Cheng et al., 2009; McAdow et al., 2011). The complexity of the *in vivo* setting hinders the exploration of bacterial gene expression in the host environment (Jenkins et al., 2015). Many studies have applied *in vitro* systems to mimic *in vivo* environments, including environments defined by low oxygen and nutrient levels (Lory et al., 1996; Shemesh et al., 2007; Malachowa et al., 2011). Different virulence determinants are required in the infection of different body sites by *S. aureus*. The factors required in the formation of BA through *S. aureus* infection, however, have not been elucidated to date. In this study, we specifically aimed to characterize the roles of 42 putative virulence determinants in BA formation through *S. aureus* infection and screen potential therapeutic targets. We analyzed and compared the virulence gene expression of two *S. aureus* strains in mouse models of BA, EC, and BM. We selected the EC and BM models to represent the stepwise development of BA through infection by skin-colonizing *S. aureus* after brain injury or by hematogenously disseminated *S. aureus*.

The *in vivo* environment imposes immune stress and metal cation limitations on bacterial pathogens (Jenkins et al., 2015). Our RT-qPCR results revealed that the expression levels of all virulence genes, except for those of the *clfA* and *essA* genes of the Newman strain and the *icaD* gene of the XQ strain, increased during the transition from EC to BA (Figure 2). The variations in the expression patterns of these genes coincide with the transformation of *S. aureus* from a commensal species to a pathogenic species and reflect the strategies used by *S. aureus* to counteract the host immune system and facilitate metal cation acquisition. During the transition from BM to BA, the expression levels of 78.6% of the selected virulence genes were downregulated. The *isdA, isdB, isdC, sasF, sdrC, hla, pvlF, aur*, and *spa* (Figure 3) genes of at least one strain were upregulated during BM to BA transition and likely play important roles in BA formation through *S. aureus* infection. Thus, we subjected these 9 genes and other 14 genes with expression levels that were significantly upregulated during EC to BA transition (Figure 2) to TaqMan qPCR analysis and isogenic mutant experiments. We demonstrated for the first time that the five enriched genes *isdA, isdC, lgt, hla*, and *spa* are required in BA formation through *S. aureus* infection.

Bacterial adherence is the first and one of the most important steps in host tissue colonization and disease development (Moreillon et al., 1995; McAdow et al., 2011; Kwiecinski et al., 2014; Walker et al., 2017). ClfA and ClfB adhesins are required during the early stage of renal abscess formation by *S. aureus* (Cheng et al., 2009). We characterized the expression patterns of typical *S. aureus* adhesin genes, including *aaa, fnbA, fnbB, sasF, sdrC*, and the extracellular adhesin genes *clfA, clfB, emp*, and *eap* (Cheng et al., 2009; Zecconi and Scali, 2013), in mouse models of BA, EC, and BM and found that none of these genes were enriched during BA formation (Figure 2A). This result may be attributed to our use of *S. aureus*-encapsulated agarose beads to establish the BA model (Baldwin and Kielian, 2004; Bloch et al., 2005). Agarose may have protected *S. aureus* cells from environmental pressures during the early colonization stage. Notably, the Newman strain lacks functional FnbAB proteins (Mulcahy and McLoughlin, 2016). The putative virulence determinant SdrC can bind the neuronal cell-adhesion molecule β-neurexin to stabilize adhesion and plays a central role in the colonization of the nasal cavity by *S. aureus* (Sudhof, 2008). Nevertheless, the deletion of *sdrC* did not impair bacterial virulence in the mouse model of BA. SasF is an important component in biofilm matrix formation by *S. aureus* during acute bone implant infection (Lei et al., 2017). It participates in skin abscess formation in mice but not in renal abscess pathogenesis (Cheng et al., 2009; Kwiecinski et al., 2014). The decrease in the brain CFUs of the Δ*sasF* mutant and the Δ*sasF*/Δ*sdrC* mutant suggests that SasF does not participate in *S. aureus*-induced BA. Notably, the results of TaqMan qPCR detection revealed that the expression levels of the *isd* genes of adhesins, which are responsible for scavenging iron from hemoproteins and transporting heme iron into *S. aureus* (Zecconi and Scali, 2013), of at least one strain were upregulated in the BA model relative to those in the BM model. In the murine BA model, the virulence of Newman mutants with *isdA* or *isdC* deletions was attenuated compared with that of wild-type strain. The *mntC* gene, which is associated with manganese transport, showed limited importance in BA formation by *S. aureus* (Figure 4). Iron content varies among different organs and tissues, and *S. aureus* responds to iron restriction in host niches through the coordinated upregulation of iron acquisition pathways (Haley and Skaar, 2012). Our data further support that cell wall-anchored Isd proteins play important roles in the development of BA through *S. aureus* infection (Mazmanian et al., 2003; Haley and Skaar, 2012). We attempted to identify the potential role of the *lgt* gene in BA formation. The *lgt* gene is a member of the toxin group and encodes diacylglyceryl transferase, which contributes to the maturation of lipoproteins (Nguyen and Gotz, 2016). Our TaqMan qPCR detection results showed that the expression levels of the *lgt* genes of both strains were upregulated in the BA model relative to those in the EC model and were slightly downregulated in the BA model relative to those in the BM model (Figure 4). The bacterial loads of the *lgt* deletion mutant significantly decreased in the mouse brain (*P* < 0.05). The BA volume of mice infected with the *lgt* deletion mutant also significantly decreased (*P* < 0.0001, Figure 5). We speculated that Lgt influences *S. aureus* BA formation by affecting the maturation of IsdE proteins. This hypothesis, however, requires further investigation.

*Staphylococcus aureus* produces numerous pathogenic toxins during infection (Zecconi and Scali, 2013). Unsurprisingly, the expression levels of several toxin genes of both strains increased in the BA model relative to those in the EC or BM models. These genes include the pore-forming toxin genes *hla* and *pvlF*, the protease gene *aur*, and the adenosine synthase gene *adsA* (Figure 2B, 3B, 4). Thus, we selected these toxin genes for isogenic mutant analysis. As expected, the virulence of the Δ*hla* strain was impaired, and mice infected with the Δ*hla* strain showed reduced bacterial loads and shrunken BA volume. By contrast, *aur* does not seem to be required for BA formation (Figure 5). The mechanism underlying Hla function may be attributed to its neurotoxic effects on brain cells *in vivo* and on isolated nerve terminals and cultured astrocytes *in vitro* (Dahlberg et al., 2015). The *aur* gene encodes aureolysin, a metalloprotease that can modify the cell surface proteins of *S. aureus* through adhesin cleavage and contribute to immune escape from macrophages (McAleese et al., 2001; Kubica et al., 2008). Nevertheless, in a murine skin abscess model, *aur* inactivation only negligibly attenuates virulence (Shaw, 2004). Our results suggest that Aur has a limited role in BA formation by *S. aureus*. The role of Panton–Valentine leukocidin (PVL, encoded by *pvlF* and *pvlS*) as a virulence determinant in mouse models has been questioned (Voyich et al., 2006; Hu et al., 2015). The *adsA* gene encodes AdsA that likely participates in the synthesis of immune suppressors and has a global effect on the physiological properties of *S. aureus* (Thomer et al., 2016). We were unable to obtain viable isogenic mutant Δ*pvlF* and Δ*adsA* strains given technical problems. In future work, we will determine the roles of PVL and AdsA in BA formation through *S. aureus* infection.

After identifying eight genes that encode immune evasion factors, we demonstrated that the *spa* gene, which encodes SpA, plays crucial roles in staphylococcal abscess formation. More than 90% of staphylococcus strains carry the *spa* gene (Vainio et al., 2011). SpA anchors to the bacterial cell wall and is released during bacterial growth; it can protect staphylococci from opsonophagocytic killing by reducing the antibody-mediated binding of bacteria or promote B-cell proliferation and apoptosis by acting as a B-cell superantigen that crosslinks the Fab domain of V_H_3-type B-cell receptors (Sjodahl, 1977; Cary et al., 1999). SpA variants exhibited attenuated abilities to form surface and renal abscesses (Cheng et al., 2009). In this study, we observed that the *spa* deletion mutant and *spa/hla* double mutant showed drastically attenuated virulence in BA formation (Figure 5). Given these results, SpA and Hla are promising targets for the treatment of *S. aureus* BA infections.

Brain abscesses treatment is highly complicated and requires combined medical and surgical approaches (Brouwer et al., 2014b; Patel and Clifford, 2014). The intensifying antibiotic resistance of bacteria involved in BA infections exacerbates the outcome of BA patients. Newly developed antivirulence therapeutics that neutralize bacterial toxins or block the pathways involved in virulence factor production are attractive options for the treatment of bacterial infections (Kong et al., 2016; Muhlen and Dersch, 2016). Enzymes, such as CrtN for carotenoid pigment synthesis, and toxins, such as Hla, Hlb, and PSMα, are promising targets in novel therapeutic paradigms against *S. aureus* infections (Chen et al., 2016; Kong et al., 2016; Wolfmeier et al., 2018). Neutralizing mAbs have attracted increased attention as potential antivirulence compounds since the FDA approved the use of mAbs against anthrax in 2009 (Kong et al., 2016). Several studies have shown that anti-Hla mAbs confer a high degree of protection against lethal pneumonia with *S. aureus* origins and reduce abscess formation by *S. aureus* in a dermonecrosis model (Tkaczyk et al., 2012; Foletti et al., 2013; Hua et al., 2014). Other researchers have proposed that bacterial infections could be treated effectively by a combination of antivirulence components that collectively target different types of virulence factors (Chow and Casadevall, 2012; Allen et al., 2014). In this study, we showed that treatment with Hla and SpA mAbs significantly decreased *S. aureus* loads in BA (*P* < 0.05) and reduced BA volume (*P* < 0.01) compared with treatment with PBS (Figure 6). This result suggests that mAbs therapy is an effective strategy for the treatment of BA caused by *S. aureus* infection. The selectiveness of mAbs is higher than that of antibiotics, which often fail to distinguish pathogens from nonpathogenic strains and may cause microfloral disequilibrium (Guarner and Malagelada, 2003). Despite this advantage, however, the poor penetration of the blood–brain barrier (BBB) by mAbs hinders their application in BA treatment (Flessner and Dedrick, 1998). McLoughlin et al. (2017) used fluorescein isothiocyanate-labeled dextran (40 kDa) to demonstrate that *S. aureus* infection can enhance the permeability of the BBB by decreasing vascular endothelial cadherin, claudin-5, and zonula occludens-1 levels in a dose-dependent manner. Moreover, Baldwin and Kielian (2004) reported that in the murine BA model, staphylococcal BA development results in the persistent opening of the BBB. This effect may favor the distribution of antibody drugs to the central nervous system. The *S. aureus* factors that participate in BBB opening warrant further investigation.

In conclusion, we applied *in vivo* virulence gene expression and isogenic mutant analyses to identify a set of five genes, namely, *isdA, isdC, lgt, hla*, and *spa*, that encode essential virulence factors involved in BA formation. Our results suggest that these five genes have prominent roles in the pathogenesis of staphylococcal BA. Our results also demonstrate that the genes of *S. aureus* are differentially expressed in different disease states and in response to the host environment. By applying Hla and SpA as therapeutic targets, we further demonstrated that mAbs therapy is an attractive option for the treatment of BA infections. Our results provide further insight on the management of BA, particularly BA caused by infection with *S. aureus*.

## Author Contributions

YZ and XR conceived and designed the experiments. YZ, WS, HP, YR, ZH, YY, LT, KX, and SL performed the experiments. YZ, ML, QH, XZ, JZ, XH, and XR analyzed the data. YZ, ML, and XR wrote the manuscript. All authors discussed the results and commented on the manuscript. The principal investigator is ML and XR.

## Conflict of Interest Statement

The authors declare that the research was conducted in the absence of any commercial or financial relationships that could be construed as a potential conflict of interest.

## References

[B1] Allen R. C. Popat R. Diggle S. P. Brown S. P. (2014). Targeting virulence: can we make evolution-proof drugs? *Nat. Rev. Microbiol.* 12 300–308. 10.1038/nrmicro3232 24625893

[B2] Alvarez M. L. Doné S. C. (2014). “SYBR^®^Green and TaqMan^®^ quantitative PCR arrays: expression profile of genes relevant to a pathway or a disease state,” in *RNA Mapping: Methods and Protocols*, eds Alvarez M. L. Nourbakhsh M. (New York, NY: Springer), 321–359. 10.1007/978-1-4939-1062-5_27 25055922

[B3] Baba T. Bae T. Schneewind O. Takeuchi F. Hiramatsu K. (2008). Genome sequence of *Staphylococcus aureus* strain Newman and comparative analysis of staphylococcal genomes: polymorphism and evolution of two major pathogenicity islands. *J. Bacteriol.* 190 300–310. 10.1128/jb.01000-07 17951380 PMC2223734

[B4] Baldwin A. C. Kielian T. (2004). Persistent immune activation associated with a mouse model of *Staphylococcus aureus*-induced experimental brain abscess. *J. Neuroimmunol.* 151 24–32. 10.1016/j.jneuroim.2004.02.002 15145600

[B5] Becker K. (2018). “Pathogenesis of *Staphylococcus aureus*,” in *Staphylococcus aureus*, ed. Fetsch A. (Longdon: Academic Press of Elsevier), 14–38. 10.1016/B978-0-12-809671-0.00002-4

[B6] Bloch O. Papadopoulos M. C. Manley G. T. Verkman A. S. (2005). Aquaporin-4 gene deletion in mice increases focal edema associated with staphylococcal brain abscess. *J. Neurochem.* 95 254–262. 10.1111/j.1471-4159.2005.03362.x 16181429

[B7] Brouwer M. C. Coutinho J. M. van de Beek D. (2014a). Clinical characteristics and outcome of brain abscess: systematic review and meta-analysis. *Neurology* 82 806–813. 10.1212/wnl.0000000000000172 24477107

[B8] Brouwer M. C. Tunkel A. R. van de Beek D. (2014b). Brain abscess. *N. Engl. J. Med.* 371:1758. 10.1056/NEJMc1410501 25354113

[B9] Burian M. Rautenberg M. Kohler T. Fritz M. Krismer B. Unger C. (2010a). Temporal expression of adhesion factors and activity of global regulators during establishment of *Staphylococcus aureus* nasal colonization. *J. Infect. Dis.* 201 1414–1421. 10.1086/651619 20307206

[B10] Burian M. Wolz C. Goerke C. (2010b). Regulatory adaptation of *Staphylococcus aureus* during nasal colonization of humans. *PLoS One* 5:e10040. 10.1371/journal.pone.0010040 20386721 PMC2850373

[B11] Cary S. Krishnan M. Marion T. N. Silverman G. J. (1999). The murine clan V(H) III related 7183, J606 and S107 and DNA4 families commonly encode for binding to a bacterial B cell superantigen. *Mol. Immunol.* 36 769–776. 10.1016/S0161-5890(99)00085-1 10593515

[B12] Chaves-Moreno D. Wos-Oxley M. L. Jauregui R. Medina E. Oxley A. P. Pieper D. H. (2016). Exploring the transcriptome of *Staphylococcus aureus* in its natural niche. *Sci. Rep.* 6:33174. 10.1038/srep33174 27641137 PMC5027550

[B13] Chen F. Di H. Wang Y. Cao Q. Xu B. Zhang X. (2016). Small-molecule targeting of a diapophytoene desaturase inhibits *S. aureus* virulence. *Nat. Chem. Biol.* 12 174–179. 10.1038/nchembio.2003 26780405

[B14] Cheng A. G. Kim H. K. Burts M. L. Krausz T. Schneewind O. Missiakas D. M. (2009). Genetic requirements for *Staphylococcus aureus* abscess formation and persistence in host tissues. *FASRB J.* 23 3393–3404. 10.1096/fj.09-135467 19525403 PMC2747682

[B15] Chow S. K. Casadevall A. (2012). Monoclonal antibodies and toxins-a perspective on function and isotype. *Toxins* 4 430–454. 10.3390/toxins4060430 22822456 PMC3398419

[B16] Dahlberg D. Mariussen E. Goverud I. L. Tonjum T. Maehlen J. Antal E. A. (2015). Staphylococcal alpha-hemolysin is neurotoxic and causes lysis of brain cells *in vivo* and *in vitro*. *Neurotoxicology* 48 61–67. 10.1016/j.neuro.2015.03.001 25757835

[B17] Duthie E. S. Lorenz L. L. (1952). Staphylococcal coagulase: mode of action and antigenicity. *J. Gen. Microbiol.* 6 95–107. 10.1099/00221287-6-1-2-95 14927856

[B18] Flessner M. F. Dedrick R. L. (1998). Tissue-level transport mechanisms of intraperitoneally-administered monoclonal antibodies. *J. Control Release* 53 69–75. 10.1016/S0168-3659(97)00238-1 9741914

[B19] Foletti D. Strop P. Shaughnessy L. Hasa-Moreno A. Casas M. G. Russell M. (2013). Mechanism of action and in vivo efficacy of a human-derived antibody against *Staphylococcus aureus* alpha-hemolysin. *J. Mol. Biol.* 425 1641–1654. 10.1016/j.jmb.2013.02.008 23416200

[B20] Guarner F. Malagelada J. R. (2003). Gut flora in health and disease. *Lancet.* 361 512-519. 10.1016/s0140-6736(03)12489-0 12583961

[B21] Haley K. P. Skaar E. P. (2012). A battle for iron: host sequestration and *Staphylococcus aureus* acquisition. *Microbes Infect.* 14 217–227. 10.1016/j.micinf.2011.11.001 22123296 PMC3785375

[B22] Hu Q. Cheng H. Yuan W. Zeng F. Shang W. Tang D. (2015). Panton-Valentine leukocidin (PVL)-positive health care-associated methicillin-resistant *Staphylococcus aureus* isolates are associated with skin and soft tissue infections and colonized mainly by infective PVL-encoding bacteriophages. *J. Clin. Microbiol.* 53 67–72. 10.1128/jcm.01722-14 25339405 PMC4290966

[B23] Hu Q. Peng H. Rao X. (2016). Molecular events for promotion of vancomycin resistance in vancomycin intermediate *Staphylococcus aureus*. *Front. Microbiol.* 7:1601. 10.3389/fmicb.2016.01601 27790199 PMC5062060

[B24] Hua L. Hilliard J. J. Shi Y. Tkaczyk C. Cheng L. I. Yu X. (2014). Assessment of an anti-alpha-toxin monoclonal antibody for prevention and treatment of *Staphylococcus aureus*-induced pneumonia. *Antimicrob. Agents Chemother.* 58 1108–1117. 10.1128/aac.02190-13 24295977 PMC3910899

[B25] Jenkins A. Diep B. A. Mai T. T. Vo N. H. Warrener P. Suzich J. (2015). Differential expression and roles of *Staphylococcus aureus* virulence determinants during colonization and disease. *mBio* 6 e02272-14. 10.1128/mBio.02272-14 25691592 PMC4337569

[B26] Katayama Y. Baba T. Sekine M. Fukuda M. Hiramatsu K. (2013). Beta-hemolysin promotes skin colonization by *Staphylococcus aureus*. *J. Bacteriol.* 195 1194–1203. 10.1128/jb.01786-12 23292775 PMC3592002

[B27] Kielian T. Hickey W. F. (2000). Proinflammatory cytokine, chemokine, and cellular adhesion molecule expression during the acute phase of experimental brain abscess development. *Am. J. Pathol.* 157 647–658. 10.1016/s0002-9440(10)64575-0 10934167 PMC1850136

[B28] Kobayashi S. D. Malachowa N. DeLeo F. R. (2015). Pathogenesis of *Staphylococcus aureus* abscesses. *Am. J. Pathol.* 185 1518–1527. 10.1016/j.ajpath.2014.11.030 25749135 PMC4450319

[B29] Kong C. Neoh H. M. Nathan S. (2016). Targeting *Staphylococcus aureus* toxins: a potential form of anti-virulence therapy. *Toxins* 8:E72. 10.3390/toxins8030072 26999200 PMC4810217

[B30] Kubica M. Guzik K. Koziel J. Zarebski M. Richter W. Gajkowska B. (2008). A potential new pathway for *Staphylococcus aureus* dissemination: the silent survival of *S. aureus* phagocytosed by human monocyte-derived macrophages. *PLoS One* 3:e1409. 10.1371/journal.pone.0001409 18183290 PMC2169301

[B31] Kwiecinski J. Jin T. Josefsson E. (2014). Surface proteins of *Staphylococcus aureus* play an important role in experimental skin infection. *APMIS* 122 1240–1250. 10.1111/apm.12295 25051890

[B32] Lei M. G. Gupta R. K. Lee C. Y. (2017). Proteomics of *Staphylococcus aureus* biofilm matrix in a rat model of orthopedic implant-associated infection. *PLoS One* 12:e0187981. 10.1371/journal.pone.0187981 29121106 PMC5679556

[B33] Liu H. Shang W. Hu Z. Zheng Y. Yuan J. Hu Q. (2018). A novel SigB(Q225P) mutation in *Staphylococcus aureus* retains virulence but promotes biofilm formation. *Emerg. Microbes Infect.* 7:72. 10.1038/s41426-018-0078-1 29691368 PMC5915575

[B34] Lory S. Jin S. Boyd J. M. Rakeman J. L. Bergman P. (1996). Differential gene expression by *Pseudomonas aeruginosa* during interaction with respiratory mucus. *Am. J. Respir. Crit. Care. Med.* 154 S183–S186. 10.1164/ajrccm/154.4_Pt_2.S183 8876539

[B35] Lowy F. D. (1998). *Staphylococcus aureus* infections. *N. Engl. J. Med*. 339 520–532. 10.1056/nejm199808203390806 9709046

[B36] Malachowa N. Whitney A. R. Kobayashi S. D. Sturdevant D. E. Kennedy A. D. Braughton K. R. (2011). Global changes in *Staphylococcus aureus* gene expression in human blood. *PLoS One* 6:e18617. 10.1371/journal.pone.0018617 21525981 PMC3078114

[B37] Mathisen G. E. Johnson J. P. (1997). Brain abscess. *Clin. Infect. Dis* 25 763–779; quiz 780–761. 10.1086/515541 9356788

[B38] Mazmanian S. K. Skaar E. P. Gaspar A. H. Humayun M. Gornicki P. Jelenska J. (2003). Passage of heme-iron across the envelope of *Staphylococcus aureus*. *Science* 299 906–909. 10.1126/science.1081147 12574635

[B39] McAdow M. Kim H. K. Dedent A. C. Hendrickx A. P. Schneewind O. Missiakas D. M. (2011). Preventing *Staphylococcus aureus* sepsis through the inhibition of its agglutination in blood. *PLoS Pathog.* 7:e1002307. 10.1371/journal.ppat.1002307 22028651 PMC3197598

[B40] McAleese F. M. Walsh E. J. Sieprawska M. Potempa J. Foster T. J. (2001). Loss of clumping factor B fibrinogen binding activity by *Staphylococcus aureus* involves cessation of transcription, shedding and cleavage by metalloprotease. *J. Biol. Chem.* 276 29969–29978. 10.1074/jbc.M102389200 11399757

[B41] McLoughlin A. Rochfort K. D. McDonnell C. J. Kerrigan S. W. Cummins P. M. (2017). *Staphylococcus aureus*-mediated blood-brain barrier injury: an *in vitro* human brain microvascular endothelial cell model. *Cell Microbiol* 19:e12664. 10.1111/cmi.12664 27598716

[B42] Missiakas D. M. Schneewind O. (2013). Growth and laboratory maintenance of *Staphylococcus aureus*. *Curr. Protoc. Microbiol.* 28 9C.1.1–9C.1.9. 10.1002/9780471729259.mc09c01s28 23408134 PMC6211185

[B43] Moreillon P. Entenza J. M. Francioli P. McDevitt D. Foster T. J. Francois P. (1995). Role of *Staphylococcus aureus* coagulase and clumping factor in pathogenesis of experimental endocarditis. *Infect. Immun.* 63 4738–4743. 7591130 10.1128/iai.63.12.4738-4743.1995 PMC173679

[B44] Muhlen S. Dersch P. (2016). Anti-virulence strategies to target bacterial infections. *Curr. Top. Microbiol. Immunol.* 398 147–183. 10.1007/82_2015_490 26942418

[B45] Mulcahy M. E. McLoughlin R. M. (2016). Host–bacterial crosstalk determines *Staphylococcus aureus* nasal colonization. *Trends Microbiol.* 24 872–886. 10.1016/j.tim.2016.06.012 27474529

[B46] Nguyen M. T. Gotz F. (2016). Lipoproteins of gram-positive bacteria: key players in the immune response and virulence. *Microbiol. Mol. Biol. Rev.* 80 891–903. 10.1128/mmbr.00028-16 27512100 PMC4981669

[B47] Patel K. Clifford D. B. (2014). Bacterial brain abscess. *Neurohospitalist* 4 196–204. 10.1177/1941874414540684 25360205 PMC4212419

[B48] Rao Q. Zhou K. Zhang X. P. Hu Q. W. Zhu J. M. Chen Z. J. (2015). Fatal multiple organ failure in an adolescent due to community-acquired methicillin- susceptible *Staphylococcus aureus* ST121/agrIV lineage: a case report. *Rev. Med. Microbiol.* 26:1 10.1097/MRM.0000000000000050

[B49] Ruthensteiner B. Hess M. (2008). Embedding 3D models of biological specimens in PDF publications. *Microsc. Res. Tech.* 71 778–786. 10.1002/jemt.20618 18785246

[B50] Shaw L. (2004). The role and regulation of the extracellular proteases of *Staphylococcus aureus*. *Microbiology* 150 217–228. 10.1099/mic.0.26634-0 14702415

[B51] Shemesh M. Tam A. Steinberg D. (2007). Differential gene expression profiling of *Streptococcus mutans* cultured under biofilm and planktonic conditions. *Microbiology* 153 1307–1317. 10.1099/mic.0.2006/002030-0 17464045

[B52] Sjodahl J. (1977). Repetitive sequences in protein A from *Staphylococcus aureus*. Arrangement of five regions within the protein, four being highly homologous and Fc-binding. *Eur. J. Biochem.* 73 343–351. 10.1111/j.1432-1033.1977.tb11324.x 557409

[B53] Sudhof T. C. (2008). Neuroligins and neurexins link synaptic function to cognitive disease. *Nature* 455 903–911. 10.1038/nature07456 18923512 PMC2673233

[B54] Tacconelli E. Tumbarello M. Cauda R. (1998). *Staphylococcus aureus* infections. *N. Engl. J. Med.* 339 2026–2027. 9882209

[B55] Thomer L. Schneewind O. Missiakas D. (2016). Pathogenesis of *Staphylococcus aureus* bloodstream infections. *Ann. Rev. Pathol.* 11 343–364. 10.1146/annurev-pathol-012615-044351 26925499 PMC5068359

[B56] Tkaczyk C. Hua L. Varkey R. Shi Y. Dettinger L. Woods R. (2012). Identification of anti-alpha toxin monoclonal antibodies that reduce the severity of *Staphylococcus aureus* dermonecrosis and exhibit a correlation between affinity and potency. *Clin. Vaccine Immunol.* 19 377–385. 10.1128/cvi.05589-11 22237895 PMC3294626

[B57] Vainio A. Koskela S. Virolainen A. Vuopio J. Salmenlinna S. (2011). Adapting spa typing for national laboratory-based surveillance of methicillin-resistant *Staphylococcus aureus*. *Eur. J. Clin. Microbiol. Infect. Dis.* 30 789–797. 10.1007/s10096-011-1158-5 21271269

[B58] Voyich J. M. Otto M. Mathema B. Braughton K. R. Whitney A. R. Welty D. (2006). Is Panton-Valentine leukocidin the major virulence determinant in community-associated methicillin-resistant *Staphylococcus aureus* disease? *J. Infect. Dis.* 194 1761–1770. 10.1086/509506 17109350

[B59] Walker J. N. Flores-Mireles A. L. Pinkner C. L. Schreiber H. L. T. Joens M. S. Park A. M. (2017). Catheterization alters bladder ecology to potentiate *Staphylococcus aureus* infection of the urinary tract. *Proc. Natl. Acad. Sci. U.S.A.* 114 E8721–E8730. 10.1073/pnas.1707572114 28973850 PMC5642702

[B60] Weidenmaier C. Peschel A. Xiong Y. Q. Kristian S. A. Dietz K. Yeaman M. R. (2005). Lack of wall teichoic acids in *Staphylococcus aureus* leads to reduced interactions with endothelial cells and to attenuated virulence in a rabbit model of endocarditis. *J. Infect. Dis.* 191 1771–1777. 10.1086/429692 15838806

[B61] Wolfmeier H. Mansour S. C. Liu L. T. Pletzer D. Draeger A. Babiychuk E. B. (2018). Liposomal therapy attenuates dermonecrosis induced by community-associated methicillin-resistant *Staphylococcus aureus* by targeting alpha-type phenol-soluble modulins and alpha-hemolysin. *EBioMedicine* 33 211–217. 10.1016/j.ebiom.2018.06.016 29936135 PMC6085503

[B62] Yuan J. Yang J. Hu Z. Yang Y. Shang W. Hu Q. (2018). Safe staphylococcal platform for the development of multivalent nanoscale vesicles against viral infections. *Nano Lett.* 18 725–733. 10.1021/acs.nanolett.7b03893 29253342

[B63] Zecconi A. Scali F. (2013). *Staphylococcus aureus* virulence factors in evasion from innate immune defenses in human and animal diseases. *Immunol. Lett.* 150 12–22. 10.1016/j.imlet.2013.01.004 23376548

[B64] Zhang X. Hu X. Rao X. (2017). Apoptosis induced by *Staphylococcus aureus* toxins. *Microbiol. Res.* 205 19–24. 10.1016/j.micres.2017.08.006 28942840

