# Supplementary material for: Virulence Determinants Are Required for Brain Abscess Formation Through Staphylococcus aureus Infection and Are Potential Targets of Antivirulence Factor Therapy
Source: Front Microbiol. 2019 Apr 5;10:682. doi: 10.3389/fmicb.2019.00682 (PMC6460967; doi:10.3389/fmicb.2019.00682)
Supplement: Supplementary file 1 [file Table_1.docx]

***Supplementary Material***

**Virulence determinants are required for brain** **abscess formation through *Staphylococcus aureus* infection and are potential targets of antivirulence factor therapy**

**Ying Zheng^1^, Weilong Shang^1^, Huagang Peng^1^, Yifan Rao^2^, Xia Zhao^1^, Zhen Hu^1^, Yi Yang^1^, Qiwen Hu^1^, Li Tan^1^, Kun Xiong^1^, Shu Li^1^, Junmin Zhu^1^, Xiaomei Hu^1^, Renjie Zhou^3^, Ming Li^1,^*, Xiancai Rao^1,^***

^1^ Department of Microbiology, College of Basic Medical Sciences, Army Medical University, Key Laboratory of Microbial Engineering under the Educational Committee in Chongqing, Chongqing, 400038, China

^2^ Institute of Modern Biopharmaceuticals, School of Life Sciences, Southwest University, Chongqing, 400715, China

^3^ Department of Emergency, Xinqiao Hospital, Army Medical University, Chongqing, 400037, China

***Correspondence：**

M. Li

[liming@tmmu.edu.cn](mailto:liming@tmmu.edu.cn)

X. Rao

[raoxiancai@126.com](mailto:raoxiancai@126.com)


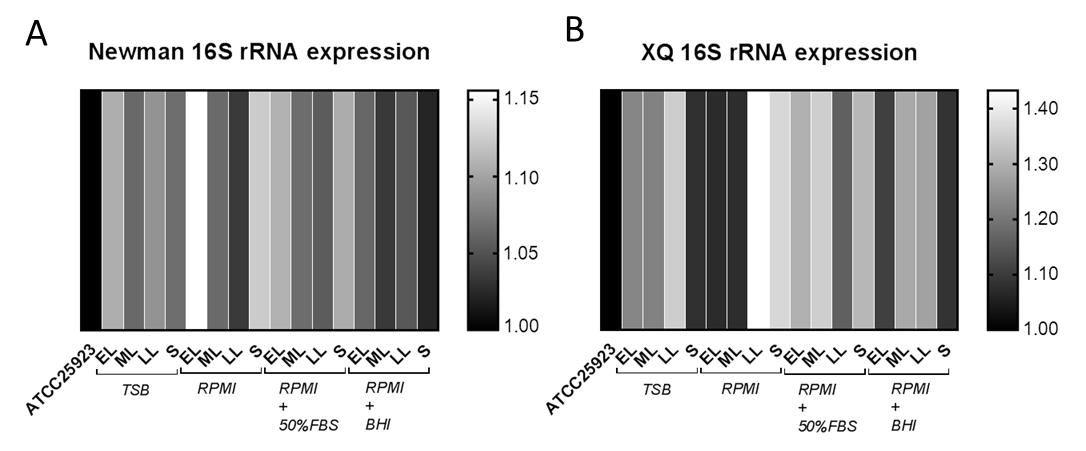


**Figure** **S1**│The expression levels of 16S rRNA gene in *S. aureus* strains Newman and XQ under varied *in vitro* growth conditions. *S. aureus* strains Newman **(A)** and XQ **(B)** were grown *in vitro* in four different growth media, trypticase soy broth (TSB), RPMI 1640 (RPMI), RPMI with 50% fetal bovine serum (RPMI + 50%FBS), and RPMI with 50% brain heart infusion (RPMI + BHI). One milliliter aliquot was removed from each culture at various growth phases [early log (EL), mid log (ML), late log (LL), and stationary (S)], and the bacterial RNA was purified and quantified as described in the Methods. The RNA concentration in each sample was diluted to 0.1 mg/ml and RT-qPCR was performed with the 16S rRNA primers. All qPCR data was compared to one sample (ATCC25923 EL in TSB media) to measure the amount of variation of the 16S rRNA internal control. None of the samples varied by more than 2-fold.


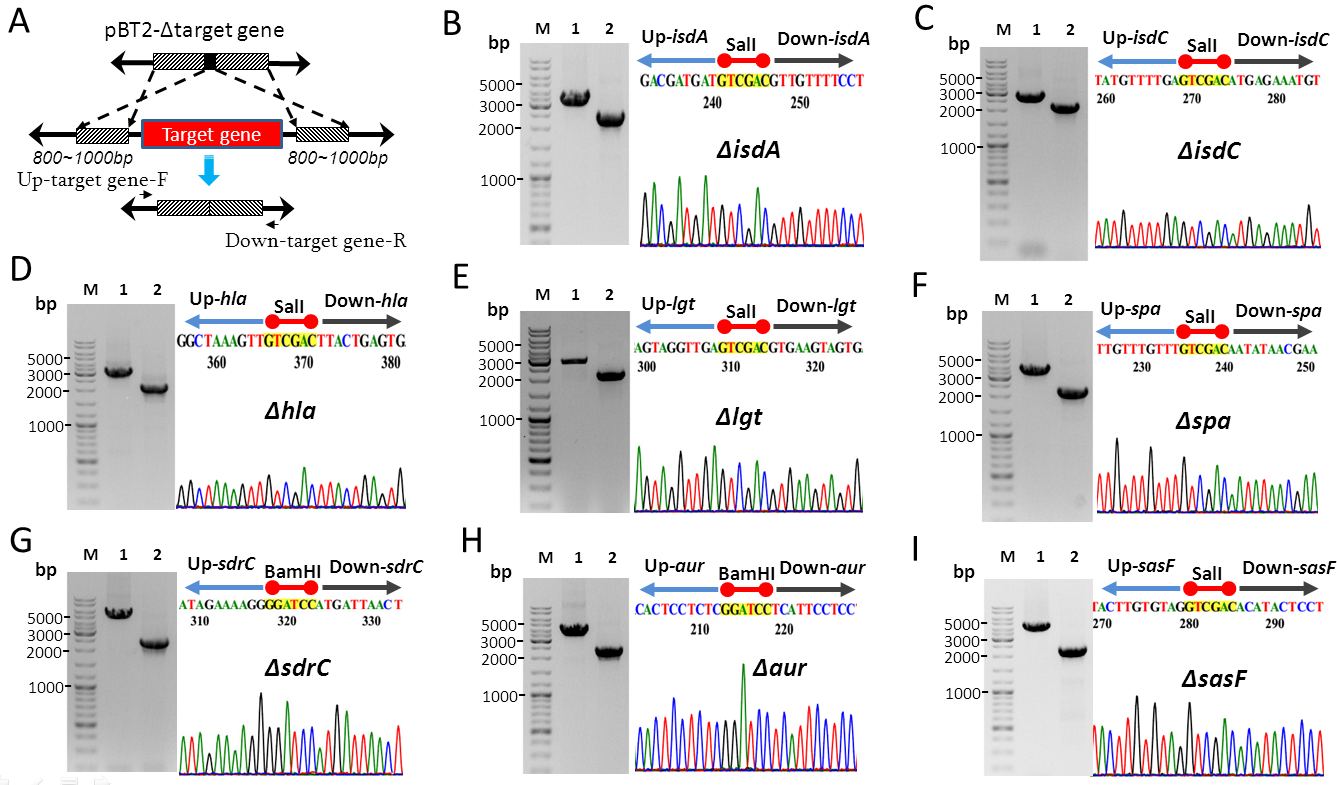


**Figure** **S2**│Identification of *S. aureus* mutants by PCR amplification and DNA sequencing. **(A)** Schematic diagram showing the markerless knock-out of the target gene in *S. aureus* Newman. To avoid the integration of exogenous DNA fragment, 800−1000 bp fragments flanking the target gene were amplified and directed fused into a shuttle vector pBT2 to generate pBT2-Δtarget gene. The deletion mutant for each target gene of interest was expected to obtain after the homologous recombination. **(B – I)** Identification of eight *S. aureus* mutants as indicated. The PCR fragments amplified using Up-target gene-F/Down-target gene-R primer pairs from the mutant genomic DNA (Lane 2 of the left panel) were shorter than that from the wild-type Newman DNA (Lane 1 of the left panel). Schematic diagrams (right panel) show correct deletions of the candidate genes, respectively. The restriction enzyme site (*Sal* I) for ligation of the left and right homologous arms was reserved for each mutant except Δ*sdrC* and Δ*aur*, which have at least one *Sal* I site in their left and/or right homologous arms and thus replaced by a *Bam*H I site for ligation.


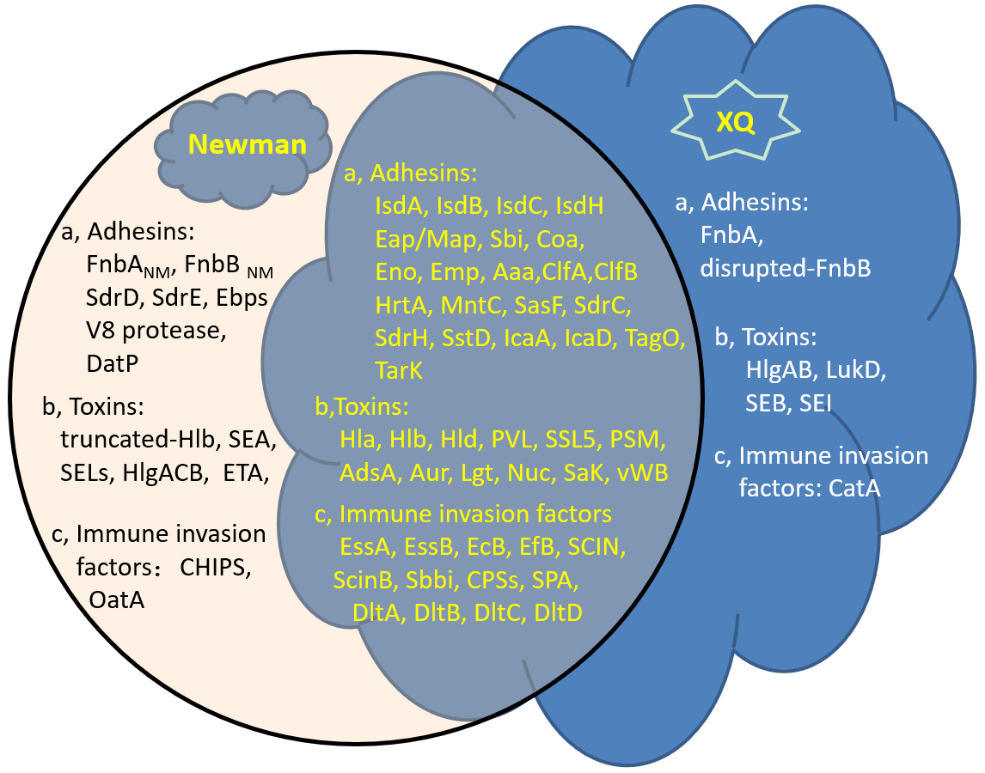


**Figure** **S3**│Venn diagram showing the virulence determinants in *S. aureus* strains Newman and XQ. The virulence determinants were obtained by searching against the complete genomes of Newman (GenBank accession no. AP009351.1) and XQ (GenBank accession no. CP013137.1) and grouped in adhesins, toxins, and immune invasion factors.


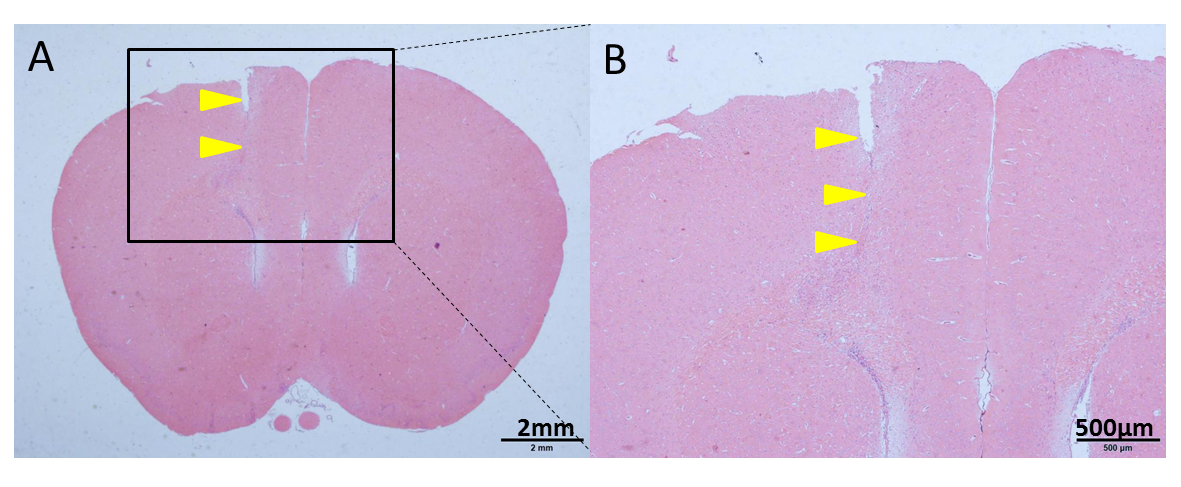


**FIGURE S4│ Pure agarose beads without *S. aureus* injected mice were unable to form brain abscesses, only a needle passway remained.** Pure agarose beads were injected into the right hemisphere of mice in the same way as described in the construction of brain abscess model. H&E staining of brain section collected from a model mouse at 5 days post injection revealed a needle passway remained at the original injection site (yellow arrowheads), failed to form any abscess. (**A**) Brain section at low magnification (20×); (**B**) Brain section at high magnification (40×).


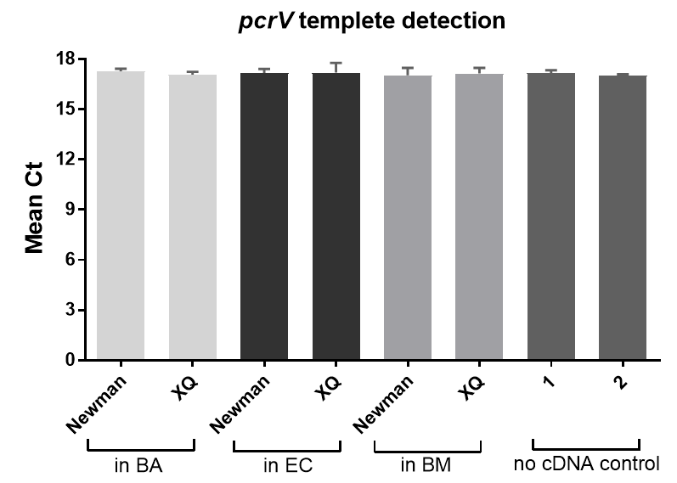


**Figure** **S5**│Impact of cDNA from different RNA source tissues on the efficiency of amplification of a test amplicon**.** *S. aureus* RNA isolated from the infected mouse tissues of brain abscess (BA), ear colonization (EC), and bacteremia (BM) was reverse transcribed into cDNA using the SuperScript III cDNA synthesis kit. Primers (**Supplementary Table S2**) were designed for quantitative detection of the *P. aeruginosa pcrV* gene. Plasmid DNA containing the *pcrV* gene was spiked into *S. aureus* cDNA samples, and no cDNA samples served as control. All samples were assayed in triplicate using SYBR^®^Green Supermix, then qPCR was conducted and analyzed using GraphPad Prism 7 software. The results are represented as mean ± SD from three independent experiments.


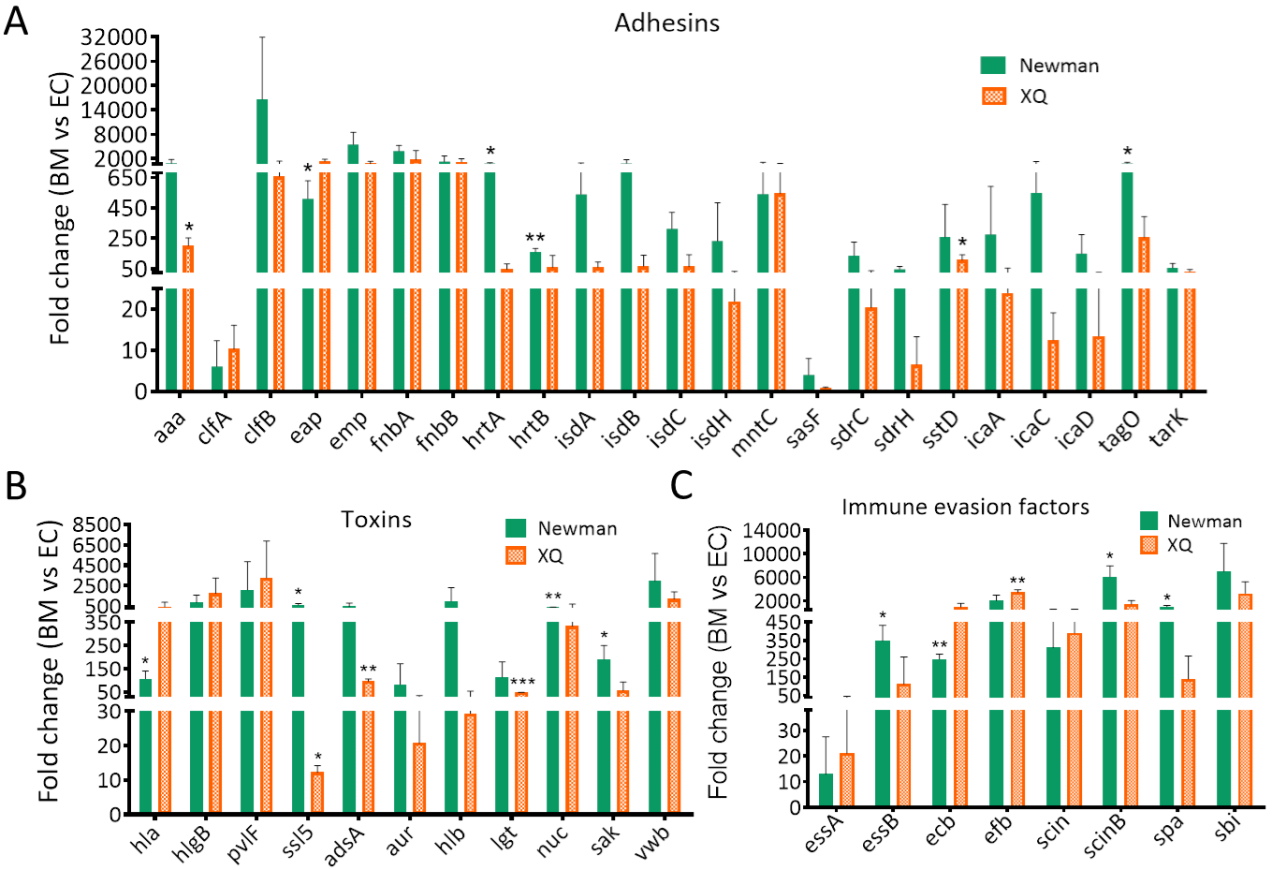


**Figure** **S6**│Variations in the expression patterns of virulence determinants in murine models of BM and EC. The transcript levels of genes were normalized to those of the 16S rRNA gene, and results for the BM model were compared with those for the EC model. The *S. aureus* strains Newman (green bars) and XQ (orange bars) were analyzed. Results are from three independent experiments with 10 animals /experiment. * *P* < 0.05, ** *P* < 0.01, *** *P* < 0.001*.* **(A)** Adhesin genes, **(B)** Toxin genes, and **(C)** Immune evasion factor genes.


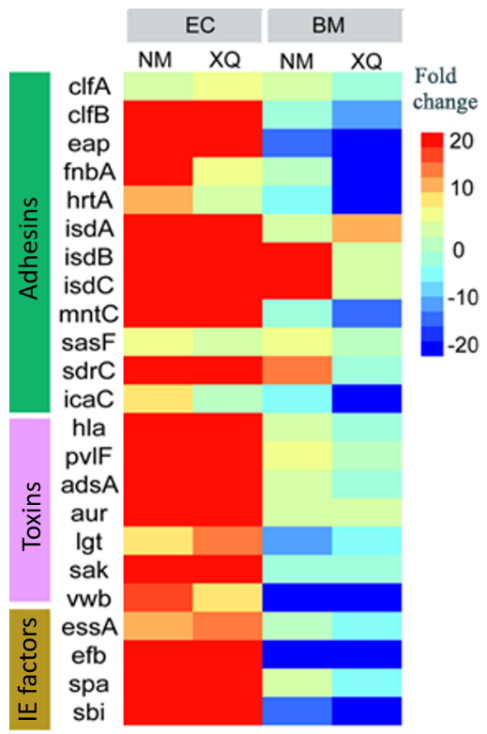


**Figure** **S7**│Heatmap showing fold change of expression levels of each virulence gene in BA model relative to EC or BM model. The transcript levels of genes were normalized to those of the 16S rRNA gene, and results for the BA model were compared with those for the EC or BM model. The *S. aureus* strains Newman (NM) and XQ were analyzed. Results are presented as mean fold of upregulation (shades of red) and downregulation (shades of blue) in three separate samples after detection by TaqMan RT-qPCR (see scale bar). Twenty-three virulence genes were divided into three groups, adhesins, toxins, and immune evasion (IE) factors as indicated.

**Figure** **S8**│Interactive three-dimensional (3D) reconstruction of BA after PBS treatment. The 3D animation document provides access to 360-degree views of the BA (in red) adjacent to the ventricle (in green). The right hemisphere was enlarged due to the mass effect of BA formation. The volume of BA is 14.9 mm^3^ (in the median of PBS control group). See attached **Supplementary Figure S8** in pdf document.

**Figure** **S9**│Interactive three-dimensional reconstruction of BA after mAbs treatment. The 3D animation document provides access to 360-degree views of the BA (in red) adjacent to the ventricle (in green). The right hemisphere was slightly enlarged due to the mass effect of BA formation. The volume of BA is 8.1 mm^3^ (in the median of mAbs treatment group). See attached **Supplementary Figure S9** in pdf document.

**Table S1**│Bacterial strains and plasmids used in this study.

| **Strain / Plasmid** | **Information** | **References** |
| --- | --- | --- |
| ***Staphylococcus aureus*** |  |  |
| Newman | NCTC 8178, ST1/*agr* I, highly virulent , extensively used in *S. aureus* animal models | Baba et al., 2008; Duthie and Lorenz, 1952 |
| XQ | ST121/*agr* IV, first isolated by our lab, highly virulent | Liu et al., 2018; Qing Rao, 2015 |
| RN4220 | Derivative of strain NCTC 8325-4, restriction deﬁcient clone host | Peng et al., 2017 |
| ***Escherichia coli*** |  |  |
| DH5α | Clone host strain | Tiangen |
| **pBT2** | E. coli - S. aureus shuttle vector for allelic exchange |  |
| pBT2-*Δaur* | pBT2 derivative for *aur* deletion in Newman | This study |
| pBT2-*Δhla* | pBT2 derivative for *hla* deletion in Newman | This study |
| pBT2-*ΔisdA* | pBT2 derivative for *isdA* deletion in Newman | This study |
| pBT2-*ΔisdC* | pBT2 derivative for *isdC* deletion in Newman | This study |
| pBT2-*Δlgt* | pBT2 derivative for *lgt* deletion in Newman | This study |
| pBT2-*ΔsasF* | pBT2 derivative for *sasF* deletion in Newman | This study |
| pBT2-*ΔsdrC* | pBT2 derivative for *sdrC* deletion in Newman | This study |
| pBT2-*Δspa* | pBT2 derivative for *spa* deletion in Newman | This study |
| **pMD19-T vector** | T-cloning vector; Amp^r^ | TaKaRa |
| pMD19-T-*pcrV* | pMD19-T vector containing the *P. aeruginosa pcrV* gene | This study |

**Table S2**│ Primers and probes used in this study

| **Gene** | **Type** | **Sequence (5'→3')** | **Reference** | |
| --- | --- | --- | --- | --- |
| **Primers and Taqman probes for RT-qPCR** | | | | |
| *16S* | F | TGAGATGTTGGGTTAAGTCCCGCA | | This study |
| rRNA | R | CGGTTTCGCTGCCCTTTGTATTGT | |  |
|  | Probe | *FAM*- ACGAGCGCAACCCTTAAGCTTAGTT -*TAMRA* | |  |
| *aaa* | F | AGGTTCTATCGCTCAAACAGATGTAG | | This study |
|  | R | TTCTGTAAGTTAAAATACCTGGTGCA | |  |
| *adsA* | F | CGACTAGCCGAAGAAAAAGGG | | This study |
|  | R | TACCGACTGCCATAGCATCATAA | |  |
|  | Probe | *FAM*-TGTCATCGGTATGGCTAAATTAAAAACA-*TAMRA* | |  |
| *aur* | F | GATGGTGTTCATGCACCTGACA | | This study |
|  | R | ATTCGTTGGCTTTACTTTCTTCG | |  |
|  | Probe | *FAM*-AGTGAAAGTACACGCAGACAAATCAGGA-*TAMRA* | |  |
| *clfA* | F | TCTGGTACGACTGTGTATCCGC | | This study |
|  | R | TGGTGGCACTTTAGCAGTTGAA | |  |
|  | Probe | *FAM*-CACCAAGCAGGTTATGTCAAACTGAAT-*TAMRA* | |  |
| *clfB* | F | TTACAGACCGAGCAAAGGCAC | | This study |
|  | R | TGCTTGTATGTGTTTTGACCTGAA | |  |
|  | Probe | *FAM*-TGATGCGAATATTAATATTGCGGATGA-*TAMRA* | |  |
| *eap* | F | TTAGCATCAACAGGTGCAAACT | | This study |
|  | R | TGATTGCATATGGAACATGGACT | |  |
|  | Probe | *FAM*-TCTGCCGCAGCTAAGCCATTAGATA-*TAMRA* | |  |
| *ecb* | F | ACGTTTGCCGGTGAATCTCAT | | This study |
|  | R | CAGCTCTTTGTGCTTTACGGTGT | |  |
| *efb* | F | GCACGTCCACAATTTAATAAACCA | | This study |
|  | R | TCAATTCGCTCTTGTAAGACCATT | |  |
|  | Probe | *FAM*-CAGCAGCGAAAACTGATGCAACTATT-*TAMRA* | |  |
| *emp* | F | GAAATCAATAATCGCGTGAATGTAG | | This study |
|  | R | GTTTGCGTAGTAATGAAGTGGTGG | |  |
| *essA* | F | GGCAAGGTTCAGACCAAATCC | | This study |
|  | R | ACGGCATCAGCAGTGCTATTC | |  |
|  | Probe | *FAM*-TCTGATTTAACACGTGCACAAGGTGA-*TAMRA* | |  |
| *essB* | F | GATCAAGCGAAACAATTAGCGG | | This study |
|  | R | CACCTTATTGGCGAACTGTCCTT | |  |
| *fnbA* | F | CAATGATCGTTGTTGGGATGG | | This study |
|  | R | TGCGTTTGACGGTTGTTCTGT | |  |
|  | Probe | *FAM*-CAAAGAAGCTGCAGCATCAGAACAA-*TAMRA* | |  |
| *fnbB* | F | ACTGAAAGTAAAGCAAGCGAAACA | | This study |
|  | R | TCCGATGGCAAATCAACTCG | |  |
| *hla* | F | GTGGTTTAGCCTGGCCTTCA | | This study |
|  | R | CGAAACATTTGCACCAATAAGG | |  |
|  | Probe | *FAM*-CCTTTAAGGTACAGTTGCAACTACCTGAT-*TAMRA* | |  |
| *hlb* | F | GGTGGGACAAAACTGAAGGTAGC | | This study |
|  | R | TGCTATCATTATCGAATCCACAACC | |  |
| *hlgB* | F | ATGTTGGCTGGGGAGTTGAA | | This study |
|  | R | GCGTATGCACTGCTTTGTCTGC | |  |
| *hrtA* | F | ATGGTGCCTCTGGTTCTGGG | | This study |
|  | R | CGTAAATCACTAGGACGATGCTGTT | |  |
|  | Probe | *FAM*-CATTGCTAACGATATTAGGCGGATTG-*TAMRA* | |  |
| *hrtB* | F | ATTGTGCTATTGAACGATAACGGAT | | This study |
|  | R | TGCTTGCTCTGCTTGATAACTCG | |  |
| *icaA* | F | TCGCACTCTTTATTGATAGTCGCT | | This study |
|  | R | GTATTCCCTCTGTCTGGGCTTG | |  |
| *icaC* | F | AGGTCAATGGTATGGCTATTTTATCG | | This study |
|  | R | ACGGTATCGTGAAACGCTGTG | |  |
|  | Probe | *FAM*-CGTTGTTATCATGCAATTCTTTATTTTGA-*TAMRA* | |  |
| *icaD* | F | ATACCCAACGCTAAAATCATCGC | | This study |
|  | R | GCAACACGTATTGTATTGATACTTTCG | |  |
| *isdA* | F | CGCAGACAGCCAACAAGTCA | | This study |
|  | R | TGAAGAGCCATCTTTTTGCACT | |  |
|  | Probe | *FAM*-CAAGTCAATGCGGCAACAGAAGCTA-*TAMRA* | |  |
| *isdB* | F | TTGAGGCCCCTACTTCTGAAAC | | This study |
|  | R | TTGGACGAGAGTTTGGTGCG | |  |
|  | Probe | *FAM*-AAGTTAAAGAAGTTAAAGCCCCTAAGGAA-*TAMRA* | |  |
| *isdC* | F | TTCTGCTTCAGGTAGTGACAAAGG | | This study |
|  | R | TGCGATTAATAATGCTAAGGATGC | |  |
|  | Probe | *FAM*-ATGGARCGACTACTGGTCAAAGTGAAT-*TAMRA* | |  |
| *isdH* | F | CTAGGCGTTGCATCGGTCA | | This study |
|  | R | GCAGGATAGTTTTTCGCAGTGTT | |  |
| *lgt* | F | AGGTGGCTTTATTGCTGGTGTT | | This study |
|  | R | CTCGTGATTCATAAAGTTACCCCA | |  |
|  | Probe | *FAM*-TAAACCCATTTCAAATTGGTGATATCGT-*TAMRA* | |  |
| *mntC* | F | CAAAGCAGTGATAAGTCAAATGGC | | This study |
|  | R | CGTTGTATAAAATAACGTCAGCGTC | |  |
|  | Probe | *FAM*-CGACGAATTCAATTTTATATGATATGGCT-*TAMRA* | |  |
| *nuc* | F | GGTGAAACCGAATACGCCTGTA | | This study |
|  | R | CTCTAGCAAGTCCCTTTTCCACTAA | |  |
| *pvlF* | F | ACACCTAAAGACAAAATGCCTGTAAC | | This study |
|  | R | AGACCAATAGCCCCAGAAACCA | |  |
|  | Probe | *FAM*-CCAGAATTTTTAGCTGTTATGTCACATGA-*TAMRA* | |  |
| *sak* | F | GAATGGGCATTAGATGCGACA | | This study |
|  | R | TGCTCTGATAAATCTGGGACAACA | |  |
|  | Probe | *FAM*-GTAGTTGAATTAGATCCAAGCGCAAAGA-*TAMRA* | | This study |
| *sasF* | F | CGAAATTAGGAAAAGCTGAAGCA | |  |
|  | R | AAGACGTGTCGCCAGTTGATG | |  |
|  | Probe | *FAM*-TTGGCTAAAGATTGGACGAATAAAGGAT-*TAMRA* | |  |
| *sbi* | F | GGGGAAGCAAAAGCGAGTG | | This study |
|  | R | TGCACGTTCTGGGTGTTCG | |  |
|  | Probe | *FAM*-ACGCAACAAACTTCAACTAAGCACCA-*TAMRA* | |  |
| *scin* | F | GGGAACTTTAGCAATCGTTTTAGC | | This study |
|  | R | CGTTTTGATATTCATTCGATGTTGG | |  |
| *scinB* | F | AAACGCATAGCAGAAGAATTAAGAAC | | This study |
|  | R | TGCAACTTTAGCATCAGCCATT | |  |
| *sdrC* | F | AATGAAAGGCCAAACAAGCAG | | This study |
|  | R | GTTGATGAGCCATTCACATTTGA | |  |
|  | Probe | *FAM*-CATCATTCAACAAGTTGCTTATCCAGAT-*TAMRA* | |  |
| *sdrH* | F | CGCTCATTTGAACCGCATG | | This study |
|  | R | TCGTCGCTGTGATTCGTTTTTA | |  |
| *spa* | F | GCCAAAGTGCTAACCTATTGTCAG | | This study |
|  | R | GGGTCATCTTTTAGGCTTTGGA | |  |
|  | Probe | *FAM*-AGTTAAATGAATCTCAAGCACCGAAAGC-*TAMRA* | |  |
| *ssl5* | F | GCATCAAACTGTAAATGCGAGTG | | This study |
|  | R | GTAATGCTTGCCACCTTTGCTAT | |  |
| *sstD* | F | GCGACGAAACCCGAAGTAATC | | This study |
|  | R | AAGTTCTTTTCATCTGCACCAACA | |  |
| *tagO* | F | GTCAAATTGCCGCTGCCTTAG | | This study |
|  | R | GCCAAACCATCGAGTCCATCA | |  |
| *tarK* | F | CAACAGGTGTACCACGTACTGATGT | | This study |
|  | R | CCTCTAAATGTCGGTGCGAATAG | |  |
| *vwb* | F | AATTTGGGAAAGTAATCGTGCG | | This study |
|  | R Probe | AGTTTGGAAAGGACCATATTAAATCA *FAM*-TGCAGTGGTTTCTGGGGAGAAGAAT-*TAMRA* | |  |
| *pcrV* | F  R | CAGAGCGGGGAACTCAAGGG  CGTTGAGCAGGGTGGTCTTC | | This study |

**Primers for isogenic mutant construction using pBT2**

| Up-*aur* (*Kpn* I) | F | CGGGGTACCTAGACAATCTTCAAACGCTT | This study |
| --- | --- | --- | --- |
| Up-*aur* (*Bam*H I ) | R | AAAGGATCCGAGAGGAGTGTGAGGGTTGT |  |
| Down-*aur* (*Bam*H I ) | F | ACCGGATCCTCATTCCTCCTGAAATCTTA | This study |
| Down-*aur* (*Sal* I ) | R | GCCGTCGACTGTGTAGCAGCAACATTAG |  |
| Up-*hla* (*Bam*H I) | F | AACGGATCCGGATAATCGACGTAAGAAGA | This study |
| Up- *hla* ( *Sal* I ) | R | CACGTCGACAACTTTAGCCGATAACTTCAG |  |
| Down- *hla* ( *Sal* I ) | F | AACGTCGACTTACTGAGTGATGATGAGTGATT | This study |
| Down- *hla*(*Hin*d III) | R | TCCAAGCTTTATGTCTTAGGCTCTATTCCTTC |  |
| Up-*isdA* (*Bam*H I) | F | ACCGGATCCCAGCTTTATAATCTTCTTCAGT | This study |
| Up- *isdA* ( *Sal* I ) | R | CACGTCGACATCATCGTCACACTCATAACTT |  |
| Down- *isdA* ( *Sal* I ) | F | AACGTCGACGTTGTTTTCCTCCTAAGGAT | This study |
| Down-*isdA*(*Hin*d III) | R | CCCAAGCTTTTAAATTTGGCTGATGGATAT |  |
| Up-*isdC* (*Bam*H I) | F | ATTGGATCCCTGCGTCAGCTAATGTAGG | This study |
| Up- *isdC* ( *Sal* I ) | R | GGCGTCGACTCAAAACATAATCCTCCTTT |  |
| Down- *isdC* ( *Sal* I ) | F | GGAGTCGACATGAGAAATGTTAAACAAATTGC | This study |
| Down- *isdC*(*Hin*d III) | R | CCCAAGCTTTACTGTCTTGTTGCACTTTATC |  |
| Up-*lgt* (*Hin*d III) | F | CCCAAGCTTggcttagagatggcaggtta | This study |
| Up- *lgt* ( *Sal* I ) | R | CGCGTCGACtcaacctactcctcactctta |  |
| Down- *lgt* ( *Sal* I ) | F | CGCGTCGACgtgaagtagtgatattttgagaa | This study |
| Down- *lgt* (*Bam*H I) | R | ACGGGATCCataatcttggtcgtcgctaac |  |
| Up-*sasF* (*Bam*H I) | F | AAAGGATCC CTGTTGTATTTGAGGCTGGAC | This study |
| Up- *sasF* ( *Sal* I ) | R | GGCGTCGACCTACACAAGTAAAGGAGAATG |  |
| Down- *sasF* ( *Sal* I ) | F | CCCGTCGACACATACTCCTTCCTCACTTAC | This study |
| Down-*sasF*(*Hin*d III) | R | TCCAAGCTTGCCCTACCATTTTCAGTGTTA |  |
| Up-*sdrC* (*Kpn* I) | F | CAAGGTACCGGTGTTGGAAAAGCTATTATG | This study |
| Up- *sdrC* (*Bam*H I ) | R | TTTGGATCCCCTTTTCTATTTGTTGCTGTC |  |
| Down-*sdrC*(*Bam*H I) | F | AAAGGATCCATGATTAACTTAACCAGGTCCA | This study |
| Down- *sdrC* (*Sal* I ) | R | ATTGTCGACTATTCAAACGTTTAGGTGCTG |  |
| Up-*spa* (*Bam*H I) | F | ACCGGATCCTGATTTATCGCCTAAAGGAATT | This study |
| Up- *spa* ( *Sal* I ) | R | GCGGTCGACAAACAAACAATACACAACGATAG |  |
| Down- *spa* ( *Sal* I ) | F | CGCGTCGACAATATAACGAATTATGTATTGCA | This study |
| Down- *spa* (*Hin*d III) | R | CTCAAGCTTCATCAGCAAGAAAACACACTTC |  |

**Table S3**│Putative virulence determinants of *S. aureus* analyzed in this study

| **Classification** | | **Genes** | **Functions and Ligands** | | **References** |
| --- | --- | --- | --- | --- | --- |
| **Adhesins** | Cell wall-anchored proteins | *aaa* | Colonization of fibrinogen and fibronectin coated surfaces | Fibrinogen; fibronectin;  vitronectin | Heilmann et al., 2005 |
|  |  | *clfA* | Interfere with phagocytosis; promote degradation of C3b;renal abscess; skin abscess; sepsis (heart involvement); survival in blood | Fibrinogen;  fibrin | Cheng et al., 2009; Hair et al., 2010; Higgins et al., 2006; Kwiecinski et al., 2014; McAdow et al., 2011; McDevitt et al., 1995; Niemann et al., 2004; Palmqvist et al., 2004 |
|  |  | *clfB* | Nasal colonization; nasal epithelial cell adhesion; catheter-associated urinary tract infection; renal abscess | Fibrinogen;  cytokeratin 10 (K10) | Cheng et al., 2009; Corrigan et al., 2009; Ni Eidhin et al., 1998; Walker et al., 2017; Walsh et al., 2008; Walsh et al., 2004; Wertheim et al., 2008 |
|  |  | *eap* | Immune modulation； renal abscess | Fibrinogen; fibronectin; prothrombin; intercellular adhesion molecule 1 | Chavakis et al., 2002; Cheng et al., 2009; Palma et al., 1999 |
|  |  | *emp* | Renal abscess | Fibronectin ; fibrinogen;  vitronectin | Cheng et al., 2009; Hussain et al., 2001 |
|  |  | *fnbA* | Colonization of heart valves; endothelial invasion; blood flow dissemination; skin abscess | Fibronectin; plasminogen; elastin | Geoghegan et al., 2013; Greene et al., 1995; Kwiecinski et al., 2014; Piroth et al., 2008; Que et al., 2005; Roche et al., 2004 |
|  |  | *fnbB* | Skin abscess | Fibronectin; elastin | Greene et al., 1995; Kwiecinski et al., 2014; Roche et al., 2004 |
|  |  | *hrtA* | Alleviates heme toxicity; attenuate liver abscess; interfere with polymorphonuclear cells recruitment | ND | Stauff and Skaar, 2009 |
|  |  | *hrtB* | Alleviates heme toxicity | ND | Stauff and Skaar, 2009 |
|  |  | *isdA* | Haem uptake and iron acquisition; renal abscess; nasal epithelial cell adhesion; innate immune evasion | Haem; fibrinogen; fibronectin; cytokeratin 10; loricrin | Cheng et al., 2009; Clarke et al., 2009; Corrigan et al., 2009; Grigg et al., 2010 |
|  |  | *isdB* | Haem uptake and iron acquisition; renal abscess | Haem; hemoglobin; integrin | Cheng et al., 2009; Grigg et al., 2010; Torres et al., 2006; Zapotoczna et al., 2013 |
|  |  | *isdC* | Haem uptake and iron acquisition; renal abscess | Haem | Cheng et al., 2009; Grigg et al., 2010 |
|  |  | *isdH* | Haem uptake and iron acquisition; promote degradation of C3b | Haem; hemoglobin | Grigg et al., 2010; Visai et al., 2009 |
|  |  | *mntC* | Manganese transportation | Manganese | Juttukonda and Skaar, 2015 |
|  |  | *sasF* | Skin abscess | ND | Kwiecinski et al., 2014 |
|  |  | *sdrC* | Nasal colonization; nasal epithelial cell adhesion | β-neurexin | Barbu et al., 2010; Corrigan et al., 2009; Jenkins et al., 2015 |
|  |  | *sdrH* | Unknown, may relate to nasal colonization | ND | Muthukrishnan et al., 2011 |
|  |  | *sstD* | Iron acquisition | ND | Beasley et al., 2011 |
|  | Extracellular sugar-based polymers | *icaA* | Polysaccharide intercellular adhesin (PIA) or N-acetyl-glucosamine (PNAG) synthesis | ND | O'Gara, 2007 |
|  |  | *icaC* | PIA/ PNAG synthesis | ND | O'Gara, 2007 |
|  |  | *icaD* | PIA/PNAG synthesis | ND | O'Gara, 2007 |
|  |  | *tagO* | WTA biosynthesis; nasal colonization | ND | Mulcahy and McLoughlin, 2016 |
|  |  | *tarK* | WTA biosynthesis; nasal colonization | ND | Mulcahy and McLoughlin, 2016 |
| **Toxins** | Pore-forming protein toxins | *hla* | Cell lysis; tissue damage; immune modulation | ADAM-10 | Kobayashi et al., 2015 |
|  |  | *hlgB* | Component of γ-hemolysin, cell lysis | ND | Alonzo and Torres, 2014; Dalla Serra et al., 2005 |
|  |  | *lukF-PV/pvlF* | Monocytes, neutrophil lysis; dermonecrosis; necrotizing pneumonia | C5aR and C5L2 | Loffler et al., 2013; Shallcross et al., 2013; Spaan et al., 2013 |
|  | Superantigens | *ssl5* | Inhibits leukocyte activation | P-selectin glycoprotein ligand 1 (PSGL-1);  G protein–coupled receptors (GPCRs) | Bestebroer et al., 2009 |
|  | Enzymatic acting toxins | *adsA* | Induce apoptosis of macrophages | AMP; ADP; AMP | Thammavongsa et al., 2013; Thammavongsa et al., 2011 |
|  |  | *aur* | Cleave complement C3; alleviated virulence in skin abscess without significance | Complement C3 | Laarman et al., 2011; Shaw, 2004 |
|  |  | *hlb* | Pneumonia; endocarditis; skin colonization; erythrocyte lysis | Sphingomyelin | Flores-Díaz et al., 2016; Katayama et al., 2013 |
|  |  | *lgt* | Lipoprotein diacylation; sepsis | Lipoproteins | Schmaler et al., 2009 |
|  |  | *nuc* | Degradation of neutrophil extracellular traps (NETs); lung infection | DNA | Berends et al., 2010 |
|  |  | *sak* | Interfere with phagocytosis; attenuate severity of sepsis | Human plasminogen; human neutrophil peptides; α-defensins | Bokarewa et al., 2006; Kwieciński et al., 2010 |
|  |  | *vwb* | Sepsis (heart involvement) | Von Willebrand factor | Bjerketorp et al., 2002; McAdow et al., 2011 |
| **Immune evasion factors** | Related to leukocyte migration and phagocytic activity | *essA* | Kidney abscess | ND | Burts et al., 2005 |
|  |  | *essB* | Kidney abscess | ND | Burts et al., 2005 |
|  | Related to complement | *ecb* | Complement inhibition; host tissues persistence;  renal abscess formation;  block neutrophil migration into lungs | C3b-containing convertases | Jongerius et al., 2007; Jongerius et al., 2012 |
|  |  | *efb* | Complement inhibition ; host tissues persistence;  renal abscess formation;  block neutrophil migration into lungs | Fibrinogen; C3b-containing convertases | Jongerius et al., 2007; Jongerius et al., 2012; Lee et al., 2004; Palma et al., 1998 |
|  |  | *scin* | Complement inhibition; inhibit phagocytosis | C3 convertase | Jongerius et al., 2007; Rooijakkers et al., 2005 |
|  |  | *scinB* | Complement inhibition; inhibits phagocytosis | C3 convertases | Jongerius et al., 2007 |
|  | Related to immunoglobulins and opsonization | *sbi* | Complement inhibition; inhibit neutrophils killing;  avoid opsonophagocytosis | IgG;  C3b; Factor H | Haupt et al., 2008; Smith et al., 2011 |
|  |  | *spa* | Retrain opsonophagocytosis; B cell superantigen; promote inflammation; renal abscess; pneumonia; skin abscess; nasal colonization | IgG; IgM; Von Willebrand factor | Cary et al., 1999; Cheng et al., 2009; Gomez et al., 2004; Hartleib et al., 2000; Kwiecinski et al., 2014; Mulcahy and McLoughlin, 2016; Sjodahl, 1977 |

ND = not determined.

**References**

Alonzo, F., 3rd, and V.J. Torres. (2014). The bicomponent pore-forming leucocidins of *Staphylococcus aureus*. *Microbiol. Mol. Biol. Rev.* 78: 199–230. [doi:org/10.1128/mmbr.00055-13](http://doi.org/10.1128/mmbr.00055-13)

Baba, T., T. Bae, O. Schneewind, F. Takeuchi, and K. Hiramatsu. (2008). Genome sequence of *Staphylococcus aureus* strain Newman and comparative analysis of staphylococcal genomes: polymorphism and evolution of two major pathogenicity islands. *J. Bacteriol.* 190: 300–310. doi:org/10.1128/jb.01000-07

Barbu, E.M., V.K. Ganesh, S. Gurusiddappa, R.C. Mackenzie, T.J. Foster, T.C. Sudhof, et al. (2010). β-Neurexin is a ligand for the *Staphylococcus aureus* MSCRAMM SdrC. *PLoS Pathog.* 6: e1000726. doi:org/10.1371/journal.ppat.1000726

Beasley, F.C., C.L. Marolda, J. Cheung, S. Buac, and D.E. Heinrichs. (2011). *Staphylococcus aureus* transporters Hts, Sir, and Sst capture iron liberated from human transferrin by Staphyloferrin A, Staphyloferrin B, and catecholamine stress hormones, respectively, and contribute to virulence. *Infect. Immun.* 79: 2345–2355. doi:org/10.1128/iai.00117-11

Berends, E.T., A.R. Horswill, N.M. Haste, M. Monestier, V. Nizet, and M. von Kockritz-Blickwede. (2010). Nuclease expression by *Staphylococcus aureus* facilitates escape from neutrophil extracellular traps. *J. Innate. Immun.* 2: 576–586. doi:org/10.1159/000319909

Bestebroer, J., K.P. van Kessel, H. Azouagh, A.M. Walenkamp, I.G. Boer, R.A. Romijn, et al. (2009). Staphylococcal SSL5 inhibits leukocyte activation by chemokines and anaphylatoxins. *Blood.* 113: 328–337. doi:org/10.1182/blood-2008-04-153882

Bjerketorp, J., M. Nilsson, A. Ljungh, J.I. Flock, K. Jacobsson, and L. Frykberg. (2002). A novel von Willebrand factor binding protein expressed by *Staphylococcus aureus*. *Microbiology.* 148: 2037–2044. doi:org/10.1099/00221287-148-7-2037

Bokarewa, M.I., T. Jin, and A. Tarkowski. (2006). *Staphylococcus aureus*: Staphylokinase. *Int. J. Biochem. Cell Biol.* 38: 504–509. doi:org/10.1016/j.biocel.2005.07.005

Burts, M.L., W.A. Williams, K. DeBord, and D.M. Missiakas. (2005). EsxA and EsxB are secreted by an ESAT-6-like system that is required for the pathogenesis of *Staphylococcus aureus* infections. *Proc. Natl. Acad. Sci. USA.* 102: 1169–1174. doi:org/10.1073/pnas.0405620102

Cary, S., M. Krishnan, T.N. Marion, and G.J. Silverman. (1999). The murine clan V(H) III related 7183, J606 and S107 and DNA4 families commonly encode for binding to a bacterial B cell superantigen. *Mol. Immunol.* 36: 769–776.

Chavakis, T., M. Hussain, S.M. Kanse, G. Peters, R.G. Bretzel, J.I. Flock, et al. (2002). *Staphylococcus aureus* extracellular adherence protein serves as anti-inflammatory factor by inhibiting the recruitment of host leukocytes. *Nat. Med.* 8: 687–693. doi:org/10.1038/nm728

Cheng, A.G., H.K. Kim, M.L. Burts, T. Krausz, O. Schneewind, and D.M. Missiakas. (2009). Genetic requirements for *Staphylococcus aureus* abscess formation and persistence in host tissues. *FASEB J.* 23: 3393–3404. doi:org/10.1096/fj.09-135467

Clarke, S.R., G. Andre, E.J. Walsh, Y.F. Dufrene, T.J. Foster, and S.J. Foster. (2009). Iron-regulated surface determinant protein A mediates adhesion of *Staphylococcus aureus* to human corneocyte envelope proteins. *Infect. Immun.* 77: 2408–2416. doi:org/10.1128/iai.01304-08

Corrigan, R.M., H. Miajlovic, and T.J. Foster. (2009). Surface proteins that promote adherence of *Staphylococcus aureus* to human desquamated nasal epithelial cells. *BMC Microbiol*. 9: 22. doi:org/10.1186/1471-2180-9-22

Dalla Serra, M., M. Coraiola, G. Viero, M. Comai, C. Potrich, M. Ferreras, et al. (2005). *Staphylococcus aureus* bicomponent gamma-hemolysins, HlgA, HlgB, and HlgC, can form mixed pores containing all components. *J. Chem. Inf. Model*. 45: 1539–1545. doi:org/10.1021/ci050175y

Duthie, E.S., and L.L. Lorenz. (1952). Staphylococcal coagulase: mode of action and antigenicity. *J. Gen. Microbiol.* 6:95–107. doi:org/10.1099/00221287-6-1-2-95

Flores-Díaz, M., L. Monturiol-Gross, C. Naylor, A. Alape-Girón, and A. Flieger. (2016). Bacterial sphingomyelinases and phospholipases as virulence factors. *Microbio.l Mol. Biol. Rev.* 80: 597–628. doi:org/10.1128/mmbr.00082-15

Geoghegan, J.A., I.R. Monk, J.P. O'Gara, and T.J. Foster. (2013). Subdomains N2N3 of fibronectin binding protein A mediate *Staphylococcus aureus* biofilm formation and adherence to fibrinogen using distinct mechanisms. *J. Bacteriol.* 195: 2675–2683. doi:org/10.1128/jb.02128-12

Gomez, M.I., A. Lee, B. Reddy, A. Muir, G. Soong, A. Pitt, et al. (2004). *Staphylococcus aureus* protein A induces airway epithelial inflammatory responses by activating TNFR1. *Nat. Med.* 10: 842–848. doi:org/10.1038/nm1079

Greene, C., D. McDevitt, P. Francois, P.E. Vaudaux, D.P. Lew, and T.J. Foster. (1995). Adhesion properties of mutants of *Staphylococcus aureus* defective in fibronectin-binding proteins and studies on the expression of fnb genes. *Mol. Microbiol.* 17: 1143–1152.

Grigg, J.C., G. Ukpabi, C.F. Gaudin, and M.E. Murphy. (2010). Structural biology of heme binding in the *Staphylococcus aureus* Isd system. *J. Inorg. Biochem.* 104: 341–348. doi:org/10.1016/j.jinorgbio.2009.09.012

Hair, P.S., C.G. Echague, A.M. Sholl, J.A. Watkins, J.A. Geoghegan, T.J. Foster, et al. (2010). Clumping factor A interaction with complement factor I increases C3b cleavage on the bacterial surface of *Staphylococcus aureus* and decreases complement-mediated phagocytosis. *Infect. Immun.* 78: 1717–1727. doi:org/10.1128/iai.01065-09

Hartleib, J., N. Kohler, R.B. Dickinson, G.S. Chhatwal, J.J. Sixma, O.M. Hartford, et al. (2000). Protein A is the von Willebrand factor binding protein on *Staphylococcus aureus*. *Blood.* 96: 2149–2156.

Haupt, K., M. Reuter, J. van den Elsen, J. Burman, S. Halbich, J. Richter, et al. (2008). The *Staphylococcus aureus* protein Sbi acts as a complement inhibitor and forms a tripartite complex with host complement Factor H and C3b. *PLoS Pathog.* 4: e1000250. doi:org/10.1371/journal.ppat.1000250

Heilmann, C., J. Hartleib, M.S. Hussain, and G. Peters. (2005). The multifunctional *Staphylococcus aureus* autolysin *aaa* mediates adherence to immobilized fibrinogen and fibronectin. *Infect. Immun.* 73: 4793–4802. doi:org/10.1128/iai.73.8.4793-4802.2005

Higgins, J., A. Loughman, K.P. van Kessel, J.A. van Strijp, and T.J. Foster. (2006). Clumping factor A of *Staphylococcus aureus* inhibits phagocytosis by human polymorphonuclear leucocytes. *FEMS Microbiol. Lett.* 258: 290–296. doi:org/10.1111/j.1574-6968.2006.00229.x

Hussain, M., K. Becker, C. von Eiff, J. Schrenzel, G. Peters, and M. Herrmann. (2001). Identification and characterization of a novel 38.5-kilodalton cell surface protein of *Staphylococcus aureus* with extended-spectrum binding activity for extracellular matrix and plasma proteins. *J. Bacteriol.* 183: 6778–6786. doi:org/10.1128/jb.183.23.6778-6786.2001

Jenkins, A., B.A. Diep, T.T. Mai, N.H. Vo, P. Warrener, J. Suzich, et al. (2015). Differential expression and roles of *Staphylococcus aureus* virulence determinants during colonization and disease. *MBio.* 6: e02272–02214. doi:org/10.1128/mBio.02272-14

Jongerius, I., J. Kohl, M.K. Pandey, M. Ruyken, K.P. van Kessel, J.A. van Strijp, et al. (2007). Staphylococcal complement evasion by various convertase-blocking molecules. *J. Exp. Med*. 204: 2461–2471. doi:org/10.1084/jem.20070818

Jongerius, I., M. von Kockritz-Blickwede, M.J. Horsburgh, M. Ruyken, V. Nizet, and S.H. Rooijakkers. (2012). *Staphylococcus aureus* virulence is enhanced by secreted factors that block innate immune defenses. *J. Innate Immun.* 4: 301–311. doi:org/10.1159/000334604

Juttukonda, L.J., and E.P. Skaar. (2015). Manganese homeostasis and utilization in pathogenic bacteria. *Mol. Microbiol.* 97: 216–228. doi:org/10.1111/mmi.13034

Katayama, Y., T. Baba, M. Sekine, M. Fukuda, and K. Hiramatsu. (2013). Beta-hemolysin promotes skin colonization by *Staphylococcus aureus*. *J. Bacteriol.* 195: 1194–1203. doi:org/10.1128/jb.01786-12

Kobayashi, S.D., N. Malachowa, and F.R. DeLeo. (2015). Pathogenesis of *Staphylococcus aureus* abscesses. *Am. J. Pathol.* 185: 1518–1527. doi:org/10.1016/j.ajpath.2014.11.030

Kwiecinski, J., T. Jin, and E. Josefsson. (2014). Surface proteins of *Staphylococcus aureus* play an important role in experimental skin infection. *APMIS.* 122: 1240–1250. doi:org/10.1111/apm.12295

Kwieciński, J., E. Josefsson, J. Mitchell, J. Higgins, M. Magnusson, T. Foster, et al. (2010). Activation of plasminogen by staphylokinase reduces the severity of *Staphylococcus aureus* systemic infection. *J. Infect. Dis.* 202: 1041–1049. doi:org/10.1086/656140

Laarman, A.J., M. Ruyken, C.L. Malone, J.A.G. van Strijp, A.R. Horswill, and S.H.M. Rooijakkers. (2011). *Staphylococcus aureus* metalloprotease aureolysin cleaves complement C3 to mediate immune evasion. *J. Immunol.* 186: 6445–6453. doi:org/10.4049/jimmunol.1002948

Lee, L.Y., M. Hook, D. Haviland, R.A. Wetsel, E.O. Yonter, P. Syribeys, et al. (2004). Inhibition of complement activation by a secreted *Staphylococcus aureus* protein. *J. Infect. Dis.* 190: 571–579. doi:org/10.1086/422259

Liu, H., W. Shang, Z. Hu, Y. Zheng, J. Yuan, Q. Hu, et al. (2018). A novel SigB(Q225P) mutation in *Staphylococcus aureus* retains virulence but promotes biofilm formation. *Emerg. Microbes Infect.* 7: 72. doi:org/10.1038/s41426-018-0078-1

Loffler, B., S. Niemann, C. Ehrhardt, D. Horn, C. Lanckohr, G. Lina, et al. (2013). Pathogenesis of *Staphylococcus aureus* necrotizing pneumonia: the role of PVL and an influenza coinfection. *Expert. Rev. Anti. Infect. Ther.* 11: 1041–1051. doi:org/10.1586/14787210.2013.827891

McAdow, M., H.K. Kim, A.C. Dedent, A.P. Hendrickx, O. Schneewind, and D.M. Missiakas. (2011). Preventing *Staphylococcus aureus* sepsis through the inhibition of its agglutination in blood. *PLoS Pathog.* 7: e1002307. doi:org/10.1371/journal.ppat.1002307

McDevitt, D., P. Francois, P. Vaudaux, and T.J. Foster. (1995). Identification of the ligand-binding domain of the surface-located fibrinogen receptor (clumping factor) of *Staphylococcus aureus*. *Mol. Microbiol.* 16: 895–907.

Mulcahy, M.E., and R.M. McLoughlin. (2016). Host–bacterial crosstalk determines *Staphylococcus aureus* nasal colonization. *Trends Microbiol.* 24: 872–886. doi:org/https://doi.org/10.1016/j.tim.2016.06.012

Muthukrishnan, G., G.A. Quinn, R.P. Lamers, C. Diaz, A.L. Cole, S. Chen, et al. (2011). Exoproteome of *Staphylococcus aureus* reveals putative determinants of nasal carriage. *J. Proteome Res.* 10: 2064–2078. doi:org/10.1021/pr200029r

Ni Eidhin, D., S. Perkins, P. Francois, P. Vaudaux, M. Hook, and T.J. Foster. (1998). Clumping factor B (ClfB), a new surface-located fibrinogen-binding adhesin of *Staphylococcus aureus*. *Mol. Microbiol.* 30: 245–257.

Niemann, S., N. Spehr, H. Van Aken, E. Morgenstern, G. Peters, M. Herrmann, et al. (2004). Soluble fibrin is the main mediator of *Staphylococcus aureus* adhesion to platelets. *Circulation.* 110: 193–200. doi:org/10.1161/01.cir.0000134486.93030.e7

O'Gara, J.P. (2007). *ica* and beyond: biofilm mechanisms and regulation in *Staphylococcus epidermidis* and *Staphylococcus aureus*. *FEMS Microbiol. Lett.* 270: 179–188. doi:org/10.1111/j.1574–6968.2007.00688.x

Palma, M., A. Haggar, and J.I. Flock. (1999). Adherence of *Staphylococcus aureus* is enhanced by an endogenous secreted protein with broad binding activity. *J. Bacteriol.* 181: 2840–2845.

Palma, M., D. Wade, M. Flock, and J.I. Flock. (1998). Multiple binding sites in the interaction between an extracellular fibrinogen-binding protein from *Staphylococcus aureus* and fibrinogen. *J. Biol. Chem.* 273: 13177–13181.

Palmqvist, N., J.M. Patti, A. Tarkowski, and E. Josefsson. (2004). Expression of staphylococcal clumping factor A impedes macrophage phagocytosis. *Microbes Infect.* 6: 188–195. doi:org/10.1016/j.micinf.2003.11.005

Peng, H., Q. Hu, W. Shang, J. Yuan, X. Zhang, H. Liu, et al. (2017). WalK(S221P), a naturally occurring mutation, confers vancomycin resistance in VISA strain XN108. *J. Antimicrob. Chemother.* 72: 1006–1013. doi:org/10.1093/jac/dkw518

Piroth, L., Y.A. Que, E. Widmer, A. Panchaud, S. Piu, J.M. Entenza, et al. (2008). The fibrinogen- and fibronectin-binding domains of *Staphylococcus aureus* fibronectin-binding protein A synergistically promote endothelial invasion and experimental endocarditis. *Infect. Immun.* 76: 3824–3831. doi:org/10.1128/iai.00405-08

Rao Q., K. Zhou, X.P. Zhang, Q.W. Hu, J.M. Zhu, Z.J. Chen, et al. (2015). Fatal multiple organ failure in an adolescent due to community-acquired methicillin- susceptible *Staphylococcus aureus* ST121/agrIV lineage: A case report. *Rev. Med. Microbiol.* 26: 1.

Que, Y.A., J.A. Haefliger, L. Piroth, P. Francois, E. Widmer, J.M. Entenza, B. Sinha, M. Herrmann, P. Francioli, P. Vaudaux, et al. (2005). Fibrinogen and fibronectin binding cooperate for valve infection and invasion in *Staphylococcus aureus* experimental endocarditis. *J. Exp. Med.* 201: 1627–1635. doi:org/10.1084/jem.20050125

Roche, F.M., R. Downer, F. Keane, P. Speziale, P.W. Park, and T.J. Foster. (2004). The N-terminal A domain of fibronectin-binding proteins A and B promotes adhesion of *Staphylococcus aureus* to elastin. *J. Biol. Chem.* 279: 38433–38440. doi:org/10.1074/jbc.M402122200

Rooijakkers, S.H., M. Ruyken, A. Roos, M.R. Daha, J.S. Presanis, R.B. Sim, et al. (2005). Immune evasion by a staphylococcal complement inhibitor that acts on C3 convertases. *Nat. Immunol.* 6: 920–927. doi:org/10.1038/ni1235

Schmaler, M., N.J. Jann, F. Ferracin, L.Z. Landolt, L. Biswas, F. Götz, et al. (2009). Lipoproteins in *Staphylococcus aureus* mediate inflammation by TLR2 and iron-dependent Growth *In Vivo*. *J. Immunol.* 182: 7110–7118. doi:org/10.4049/jimmunol.0804292

Shallcross, L.J., E. Fragaszy, A.M. Johnson, and A.C. Hayward. (2013). The role of the Panton-Valentine leucocidin toxin in staphylococcal disease: a systematic review and meta-analysis. *Lancet Infect. Dis.* 13: 43–54. doi:org/10.1016/s1473-3099(12)70238-4

Shaw, L. (2004). The role and regulation of the extracellular proteases of *Staphylococcus aureus*. *Microbiology.* 150: 217–228. doi:org/10.1099/mic.0.26634-0

Sjodahl, J. (1977). Repetitive sequences in protein A from *Staphylococcus aureus*. Arrangement of five regions within the protein, four being highly homologous and Fc-binding. *Eur. J. Biochem.* 73: 343–351.

Smith, E.J., L. Visai, S.W. Kerrigan, P. Speziale, and T.J. Foster. (2011). The Sbi protein is a multifunctional immune evasion factor of *Staphylococcus aureus*. *Infect. Immun.* 79: 3801–3809. doi:org/10.1128/iai.05075-11

Spaan, A.N., T. Henry, W.J.M. van Rooijen, M. Perret, C. Badiou, P.C. Aerts, et al. (2013). The staphylococcal toxin Panton-Valentine Leukocidin targets human C5a receptors. *Cell Host Microbe.* 13: 584–594. doi:org/10.1016/j.chom.2013.04.006

Stauff, D.L., and E.P. Skaar. (2009). The heme sensor system of *Staphylococcus aureus*. *Contrib. Microbiol.* 16:120–135. doi:org/10.1159/000219376

Thammavongsa, V., D.M. Missiakas, and O. Schneewind. (2013). *Staphylococcus aureus* degrades neutrophil extracellular traps to promote immune cell death. *Science.* 342: 863–866. doi:org/10.1126/science.1242255

Thammavongsa, V., O. Schneewind, and D.M. Missiakas. (2011). Enzymatic properties of *Staphylococcus aureus* adenosine synthase (AdsA). *BMC Biochem.* 12: 56. doi:org/10.1186/1471-2091-12-56

Torres, V.J., G. Pishchany, M. Humayun, O. Schneewind, and E.P. Skaar. (2006). *Staphylococcus aureus* IsdB is a hemoglobin receptor required for heme iron utilization. *J. Bacteriol.* 188: 8421–8429. doi:org/10.1128/jb.01335-06

Visai, L., N. Yanagisawa, E. Josefsson, A. Tarkowski, I. Pezzali, S.H. Rooijakkers, et al. (2009). Immune evasion by *Staphylococcus aureus* conferred by iron-regulated surface determinant protein IsdH. *Microbiology.* 155: 667–679. doi:org/10.1099/mic.0.025684-0

Walker, J.N., A.L. Flores-Mireles, C.L. Pinkner, H.L.t. Schreiber, M.S. Joens, A.M. Park, et al. (2017). Catheterization alters bladder ecology to potentiate *Staphylococcus aureus* infection of the urinary tract. *Proc. Natl. Acad. Sci. USA.* 114: E8721–e8730. doi:org/10.1073/pnas.1707572114

Walsh, E.J., H. Miajlovic, O.V. Gorkun, and T.J. Foster. (2008). Identification of the *Staphylococcus aureus* MSCRAMM clumping factor B (ClfB) binding site in the alphaC-domain of human fibrinogen. *Microbiology.* 154: 550–558. doi:org/10.1099/mic.0.2007/010868-0

Walsh, E.J., L.M. O'Brien, X. Liang, M. Hook, and T.J. Foster. (2004). Clumping factor B, a fibrinogen-binding MSCRAMM (microbial surface components recognizing adhesive matrix molecules) adhesin of *Staphylococcus aureus*, also binds to the tail region of type I cytokeratin 10. *J. Biol. Chem.* 279: 50691–50699. doi:org/10.1074/jbc.M408713200

Wertheim, H.F., E. Walsh, R. Choudhurry, D.C. Melles, H.A. Boelens, H. Miajlovic, et al. (2008). Key role for clumping factor B in *Staphylococcus aureus* nasal colonization of humans. *PLoS Med.* 5: e17. doi:org/10.1371/journal.pmed.0050017

Zapotoczna, M., Z. Jevnikar, H. Miajlovic, J. Kos, and T.J. Foster. (2013). Iron-regulated surface determinant B (IsdB) promotes *Staphylococcus aureus* adherence to and internalization by non-phagocytic human cells. *Cell Microbiol.* 15: 1026–1041. doi:org/10.1111/cmi.12097
