# Supplementary material for: Virulence Determinants Are Required for Brain Abscess Formation Through Staphylococcus aureus Infection and Are Potential Targets of Antivirulence Factor Therapy
Source: Front Microbiol. 2019 Apr 5;10:682. doi: 10.3389/fmicb.2019.00682 (PMC6460967; doi:10.3389/fmicb.2019.00682)

Interactive 3D reconstruction of BA after mAbs treatment

To interact with the 3D reconstruction

Translate: Hold left and right mouse-bottons and move mouse.

Rotate: Hold left mouse-button and move mouse.

Zoom: Hold right mouse-button and move mouse up or down.

Selection of preset views

The views panel which in the under-part contains several preset views.

Click to select such a view.

Selection of structures

The structures panel of model tree in the left-part contains buttons for each structure to hide or show.

Technical Notes

This PDF file is preferably viewed in Adobe Reader 9.3 or higher.

(http://www.adobe.com/downloads/)

Department of Digital Medicine,
Biomedical Engineering College,
Third Military Medicl University, Chongqing, China
http://szyxyjs.tmmu.edu.cn/index.htm

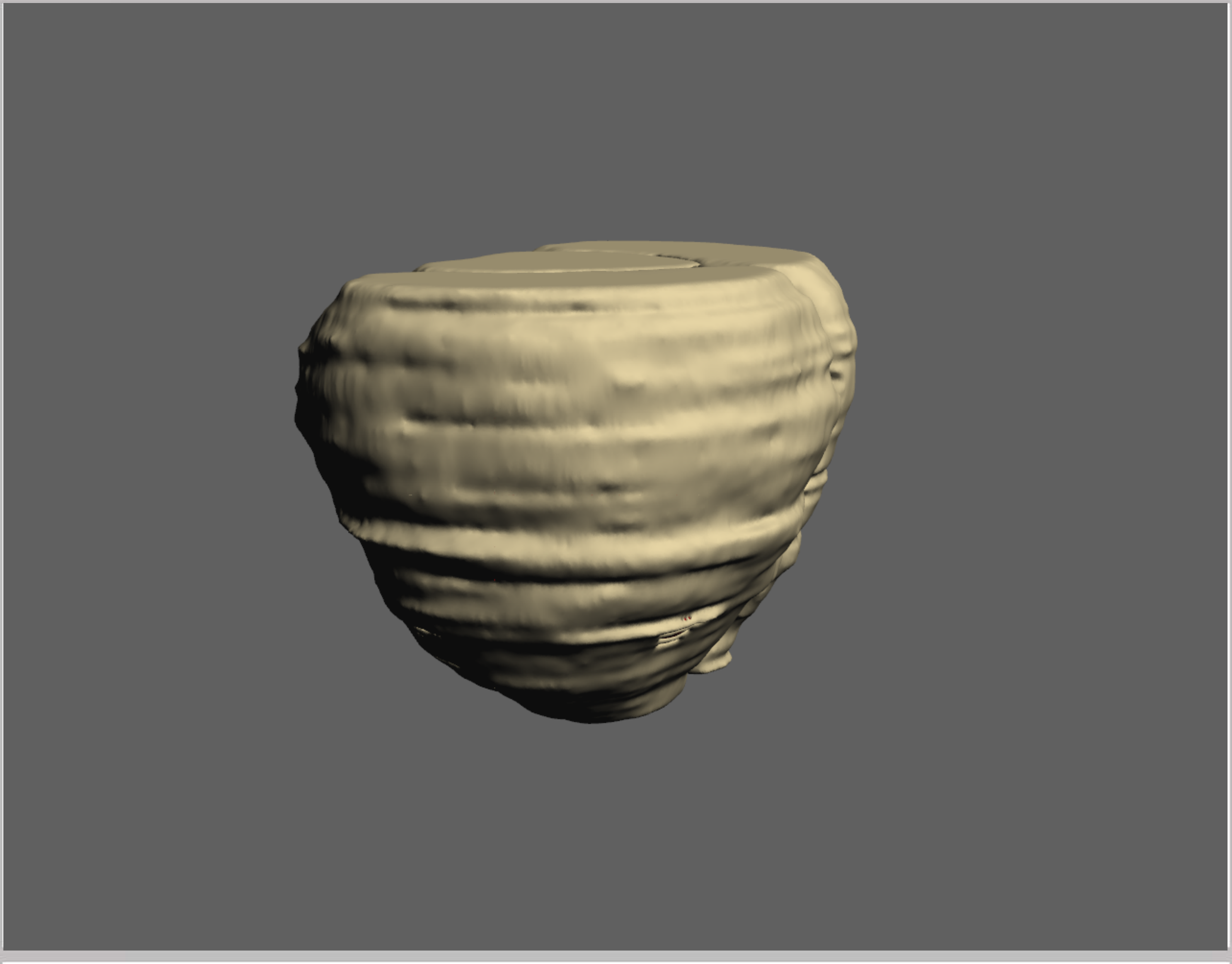

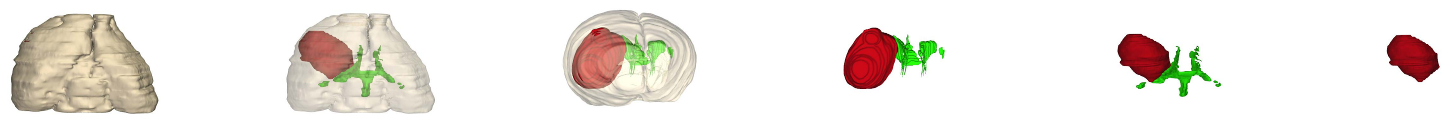

Supplement: Supplementary file 3 [file Image_2.pdf]
